# Supplementary material for: Host response during unresolved urinary tract infection alters female mammary tissue homeostasis through collagen deposition and TIMP1
Source: Nat Commun. 2024 Apr 16;15:3282. doi: 10.1038/s41467-024-47462-7 (PMC11021735; doi:10.1038/s41467-024-47462-7)
Supplement: Supplementary file 1 — Supplementary Information [file 41467_2024_47462_MOESM1_ESM.pdf]

Title: Host response during unresolved urinary tract infection alters female mammary tissue homeostasis through collagen deposition and TIMP1

Author list: Samantha Henry[1,2#], Steven Macauley Lewis[1,2#], Samantha Leeanne Cyrill[1#], Mackenzie Kate Callaway[1], Deeptiman Chatterjee[1], Amritha Varshini Hanasoge Somasundara[1,3], Gina Jones[1], Xue-Yan He[4], Giuseppina Caligiuri[1], Michael Francis Ciccone[1], Isabella Andrea Diaz[1], Amelia Biswas[1,5], Evelyn Hernandez[1], Taehoon Ha[1], John Erby Wilkinson[6], Mikala Egeblad[7], David Arthur Tuveson[1], Camila Oresco dos Santos[1]*

Affiliations: [1]Cold Spring Harbor Laboratory; Cold Spring Harbor, NY, USA. [2]Stony Brook University, Graduate Program in Genetics; Stony Brook, NY, USA. [3]CSHL School of Biological Sciences; Cold Spring Harbor, NY, USA. [4]Department of Cell Biology and Physiology. School of Medicine in St. Louis. Washington University, St. Louis., MO, USA.[5]SUNY Downstate Health Sciences University, Neural and Behavior Science; Brooklyn, NY, USA. [6]Department of Comparative Medicine, University of Washington; Seattle, WA, USA.[7]Department of Cell Biology. Department of Oncology. School of Medicine. Johns Hopkins University. Baltimore, MD, USA.

# these authors contributed equality

*corresponding author: dossanto@cshl.edu

Supplementary Methods

Antibodies. All antibodies were obtained from commercial vendors and used without further purification. *For immunofluorescence staining:* Alexa Fluor 647-conjugated anti-Cytokeratin 5 (EP1601Y, Abcam, cat# ab193895, 0.5 mg/ml, 1:200), Alexa Fluor 488-conjugated anti-β-casein (H-4, SCBT, cat# sc-166530, 0.2 mg/ml, 1:200), FITC-conjugated anti-E. coli antibody (Abcam, cat# ab30522, 5mg/ml, 1:100), anti-human/mouse myeloperoxidase antibody (R&D Systems, cat# AF3667, 0.2 mg/ml, 1:100), Anti-Histone H3 (citrulline R2 + R8 + R17) antibody (Abcam, cat# ab5103, 1 mg/ml, 1:250), Alexa Fluor 488-conjugated anti-rabbit secondary antibody (Invitrogen, cat# A21206, 2 mg/ml, 1:150, Picrosirius Red staining kit (Abcam, cat# ab150681), anti-Perilipin 1 polyclonal antibody (Thermo Fisher, cat# PA5-55046, 1:200), Alexa Fluor 488conjugated anti-Fibronectin (Abcam cat# ab237286, 1:200). *For flow cytometry:* Brilliant Violet 605-conjugated anti-CD45 antibody (30-F11, BioLegend, cat# 103140, 0.2 mg/ml, 3:200), PE/Cy7-conjugated anti-CD11b antibody (M1/70, BioLegend, cat# 101216, 0.2 mg/ml, 1:100) and, Alexa Flour 700-conjugated anti-Ly6G antibody (1A8, BioLegend, cat# 127622, 0.5 mg/ml, 3:200).

Tissue collection and processing for imaging analyses. Mammary gland (left inguinal), bladder, kidney, spleen, lungs, liver, intestines, and pancreas were harvested at the experimental endpoint and immediately fixed in 4% paraformaldehyde in 1X PBS (16% solution, Electron

Microscopy Sciences, cat# 15711) at 4°C overnight, followed by storage in 1X PBS at 4°C, until paraffin embedding. Tissue processing, paraffin embedding, sectioning, and mounting onto slides was carried out by the CSHL Shared Histology Core Facility using standard procedures. Mammary gland tissue was sectioned at 7 μm while all other tissues were mounted on to slides in ~5 μm sections. H&E staining were carried out by the CSHL Cancer Center Histology Core Facility using standard protocols.

Masson's trichrome staining, imaging and quantification. Masson's trichrome staining was carried out by the CSHL Cancer Center Histology Core Facility using standard protocols. Stained slides were scanned on an Aperio Light Field Slide Scanner (Leica Biosystems). Image acquisition was carried out with Aperio ImageScope (Leica Biosystems). Image quantification was performed in FIJI (ImageJ) using ten randomly selected images from each gland to quantify the percentage of area stained blue from each image[1,2].

Immunofluorescence (IF) staining. For the analysis of Cytokeratin 5, b-casein, *E. coli*, Perilipin, and Picrosirius Red, slide-mounted tissue sections were deparaffinized in Xylene (Sigma, cat# 534056) and antigen retrieval was conducted in Trilogy (Cell Marque, cat# 920P-10) by heating for 15 min under high pressure, followed by 5 mins without pressure. Slides were washed in 1x PBS and blocked for 4 hours in a humidified chamber with ~100 μl blocking buffer (10mM Tris-HCl pH 7.4, 100mM MgCl2, 0.5% Tween 20, 10% FBS, 5% goat serum). Tissues were then stained with fluorophore-conjugated primary antibodies in blocking buffer overnight, at 4°C, followed by three alternating washes with 1x PBS (1 min/wash) and blocking buffer (5 min/wash). Nuclear staining with DAPI (Sigma, cat# 10236276001) or propidium iodide (Invitrogen, cat# P3566, 1:1000) was carried out for 10 minutes. Slides were then mounted in ProLong Glass Antifade Mountant (Invitrogen, cat# P36980), cover-slipped and allowed to cure overnight at room temperature. For IF staining of Myeloperoxidase and citrullinated histone H3, a previously published staining protocol was used[3]. After deparaffination and rehydration of the mammary tissue, Tris-EDTA buffer (10 mM Tris Base, 1 mM EDTA, 0.05% Tween 20, pH 9.0) was used for antigen retrieval under low pressure for 8 min. The slides were then washed with cold running water and 1X PBS and subsequently blocked with Fc Receptor Blocker (Innovex Biosciences, cat# NB309) for 30 min followed by 1X Blocking Buffer (0.1% Triton-PBS containing 2.5% BSA and 5% donkey serum) for 1 hour at room temperature. The slides were then washed in 1X PBS and stained with primary antibodies in 0.5X blocking buffer at 4°C, overnight. The slides were then washed and incubated with secondary antibodies and DAPI (10 μg/ml; Invitrogen, cat# D1306) in

0.5X blocking buffer for 1 hour at room temperature. Slides were washed thrice in 1X PBS and water and then mounted and cover-slipped with ProLong™ Diamond Antifade Mountant (Invitrogen, cat# P36961).

Immunofluorescence (IF) tissue imaging and analysis. IF stained slides were imaged on Zeiss LSM710 confocal microscope with Zen 2012 SP5 software (Zeiss) or on the Zeiss Axio Observer inverted fluorescence microscope using Zen blue 2.0 software (Zeiss). For Cytokeratin 5 and βcasein analysis, IF staining was evaluated from five random field of view were taken per slide, (20x, size 600 x 600, averaging 4, 12 bit, speed 5), as previously demonstrated[4,5]. Percentage of area stained with either Cytokeratin 5 or β-casein across the whole image was quantified in FIJI. For Myeloperoxidase (Mpo) analysis, images were taken under 20x lens with 0.75x zoom-out, and integrated density (MPO to DAPI) were counted across the image after loading images into FIJI.

Perilipin staining and image analysis. For perilipin, primary antibody staining was completed overnight at 4°C in the dark and secondary antibody staining was completed for 1 hour at RT in the dark. Images of Perilipin was acquired as described above. For analysis, ten images per stained slide were acquired (40X oil, size 600 x 600, averaging 4, 12 bit, speed 5) and individual adipocytes were manually counted across the image after loading images into FIJI, as previously demonstrated[6].

Picrosirius staining, imaging and analysis. Picrosirius Red staining was conducted using a commercially available kit (Abcam, cat# ab150681) according to the manufacturer's protocol. Briefly, slides were deparaffinized (as described above for IF), rehydrated (by washing twice for 3 min. in absolute ethanol) then stained in Picrosirius Red solution for 1 hour at RT followed by dehydration and clearing (washing twice in xylene) prior to mounting. Then, stained slides were imaged under linearly polarized light on the Zeiss Axio Observer inverted fluorescence microscope using Zen blue 2.0 software (Zeiss). Five images per gland were acquired for calculation of percentage area occupied by thick and thin fibers in FIJI from the acquired images, as previously demonstrated[7].

Duct lumen area quantification and count. Ducts were counted by loading in H&E images of mammary glands (ten images per gland) into FIJI and counted manually following previously published protocols[8]. Ductal lumen area was quantified after loading H&E images utilizing the tracer and area functions in FIJI.

Analysis of collagen alignment (Second Harmonic Generation). Collagen alignment was quantified using CT-FIRE and MATLAB to extract collagen fiber information from two photon second harmonic generation images. One representative field of view was assessed per condition, and each 16X field of view was analyzed by segmenting the image into 25 ROIs of equal size, as previously demonstrated[4]. CT-FIRE was performed on each ROI, and individual fiber information was extracted to determine the degree of alignment per ROI. Relative fiber orientation was determined by finding the difference between each fiber in an ROI and the ROI's mode angle, meaning ROIs with higher alignment would show higher proportions of fibers within 15° to 30° of the mode angle (set to 0°).

Mammary tissue digestion. Mammary glands were digested into single cell suspensions using previously published protocols[9–11]. Briefly, inguinal and thoracic mammary glands were harvested, minced and digested for ~90 min at 37°C in RPMI 1640 GlutaMAX (Gibco, cat# 61870127) containing 5% FBS (Corning, cat# 35-010-CV) and 1X Collagenase-Hyaluronidase (Stem Cell Technology, cat# 07912). Digested mammary glands were centrifuged at 2000 rpm for 5 mins and the pellet was cryopreserved in 1 ml of Synth-a-Freeze™ Cryopreservation Medium (Gibco, cat# A1254201), distributed over two cryovials (Corning, cat# 430487), and stored at -80°C for short-term storage and in liquid nitrogen vapor for long-term storage. Viably frozen, digested mammary fragments were thawed in a 37°C water bath, and washed with chilled HBSS (Gibco, cat# 14175103) containing 5% FBS. Single cell suspensions were obtained by incubating the cell pellet with 3 ml of TrypLE Express (Gibco, cat# 12604013) for 3 min followed by an HBSS wash, and subsequent incubation with 1 ml of Dispase (Stem Cell Technology, cat# 07913) containing 40µl DNase I (Sigma, cat# D4263) for 2 minutes. The cell suspension was washed again in HBSS and filtered through a 100 µm cell strainer (BD Falcon, cat# c352360). Centrifugation at 2000 rpm for 5 min at room temperature was used to collect cells after each wash. The cells were resuspended in 1X MACS buffer (1X PBS with 0.5% FBS) and kept on ice prior to staining for flow cytometry.

Flow cytometry analysis. Single cells suspended in 1X MACS buffer were stained with appropriate fluorophore-conjugated primary antibodies. Staining was carried out in a 5 ml polystyrene round-bottom tube (Corning, cat# 352054) for 40 min at 4°C in the dark after which the samples were washed in 1X MACS buffer and transferred to a polystyrene round bottom tube fitted with a 35 µm cell strainer cap (Corning, cat# 352235) prior to acquisition on the BD LSRFortessa Dual SORP (BD Biosciences) using BD FACSDiva™ v9 software (BD Biosciences). Data was analyzed using FlowJo™ v10 software (BD Life Sciences).

Preparation of bacteria for infection. The uropathogenic *Escherichia coli* (UPEC) strain, UTI89 was obtained as a kind gift from the Hultgren lab at Washington University in St. Louis. UPEC inoculum was prepared as previously described[12]. Briefly, a loopful of UPEC was inoculated from a frozen glycerol stock into 20 ml of LB broth and incubated overnight at 37°C, under static conditions. The bacterial culture was then sub-cultured (1:1000) into sterile LB broth and incubated 37°C for ~18 hours. The bacteria cultures were centrifuged at 3000 g for 15 min and re-suspended in sterile 1X PBS. Bacteria were enumerated by measuring OD at 600 nm (Ultrospec 7000, GE) and titration on LB agar. The bacterial suspension was diluted in sterile 1X PBS to a concentration of ~2-8 x 10^{9} CFU/mL for mouse infection.

Mouse infections and inclusion criteria. Mice were infected by delivering active UPEC in 1X PBS (as described above) directly into the murine bladder by transurethral catheterization as described previously[12]. Briefly, catheters were prepared by sheathing a 30 gauge, half-inch needle (BD Precision Glide™, cat# 305106) with polyethylene tubing (PE10, BD Intramedic™, cat# 427401) and were sterilized under UV light for 40 min. The UPEC suspension was loaded into a 1 ml syringe with a tuberculin slip tip (BD, cat# 309659) and fitted with the prepared catheter. Mice were anesthetized by controlled isoflurane inhalation and placed in a supine position; the bladder was voided and the urogenital area was swabbed with 70% ethanol. The catheters were dipped in sterile, non-bacteriostatic lubricant (HR® Lubricating Jelly OneShot®), inserted into the mouse bladder via the urethra and 50 µl (1-4 x 10^{8} CFU) of the UPEC suspension was injected into the bladder slowly to minimize vesicoureteral reflux. For controls, mice were administered 50 µl of sterile 1X PBS. For infections in nulliparous mice, 1X PBS (control) or UPEC suspension (UTI) was administered to 7-12 weeks old mice and end-point analyses were conducted at 72 hours or 2 weeks post-infection (p.i.). For infections during lactation (PLI model), mice at lactation day 10 were administered 1X PBS or UPEC suspension. Pups were weaned at 72 hours p.i. to synchronize the initiation of involution. End-point analyses were conducted at involution day 10 (~2 weeks p.i.). Throughout all experiments, mice with bacterial titers above 5 x 10^{4} CFU/mL were considered actively infected, and those dropping below this threshold were considered resolved. Animals were tested for CFUs at 48 hours p.i. and endpoint (2 weeks p.i.) and only those actively infected, were analyzed (unless treated with TMS, as described below). At endpoint of 2 weeks p.i., 25/58 nulliparous and 7/15 PLI animals retained infection in our studies.

Urine collection and bacterial titers. Mice were restrained by scruffing and the urogenital surface was cleaned by swabbing with 70% ethanol. Free-catch urine samples were collected, by gentle palpation of the bladder, into a clean, sterile petri dish. Urine samples were collected before

UPEC infection, at 48 hours p.i., and at the experimental endpoint. Urine samples were serially diluted, 10-fold, in sterile 1X PBS and spot-plated on LB agar to estimate bacterial counts[12]. Mice with bacterial titers greater than 5 x 10^4 CFU/mL were classified as infected.

Antibiotic treatment. EQUISUL-SDT antimicrobial oral suspension (Covetrus, cat# 048889) containing Trimethoprim-Sulfamethoxazole (TMS) was added to MediGel® Sucralose cups (2 oz, ClearH_2O), at a concentration of 0.48 mg/ml. The TMS cups were placed in the animal cages as the sole source of hydration for the duration of the experiment starting 48 hours p.i.. Urine samples from TMS-treated mice were collected and titrated on LB agar plates to assess UTI resolution.

Collection of blood cells and plasma. Blood was collected by terminal cardiac bleeds into EDTA-containing MiniCollect® tubes (Greiner Bio-one, cat# 450475). Plasma was separated from blood cells, by centrifugation at 5000 rpm for 15 min at 4°C, aliquoted and stored at -20°C. The remaining cell pellet was subjected to two rounds of RBC lysis (10 min each) in 1X RBC lysis buffer (BioLegend, cat# 420301) and fixed in 2% PFA for 15-20 min on ice. The fixed cells were washed and resuspended in 1X MACs buffer and stored at 4°C until staining for flow cytometry.

Cytokine array and ELISAs. The Proteome Profiler Mouse Cytokine Array Kit, Panel A (R&D Systems #ARY006) was used to profile 40 cytokines and chemokines in plasma and was performed according to manufacturer's instructions. 100 µl of plasma from two mice in each condition were pooled for the assay. Chemiluminescent signal was captured on to an autoradiogram (Lab Scientific, cat# XARALF2025) and developed using a Mini-Med 90, Automatic Film Processor (AFP Manufacturing). The developed films were digitalized by scanning with a Perfection 2450 photo scanner (Epson). The mean pixel intensity of each spot was then measured using FIJI ImageJ[13,14]. ELISAs for detecting corticosterone (Tecan, product# RE52211), Timp1 (Invitrogen, cat# EMTIMP1), and estradiol (DRG International, cat# EIA2693) levels were carried out with commercially available kits according to manufacturer's instructions. Colorimetric readouts were obtained using the SpectraMax i3x Multi-Mode Microplate Detection Platform with SoftMax Pro software (Molecular Devices). Each sample was assayed in duplicate and reported as an average after quantifying the mean density of signal in FIJI. Values are reported relative to the positive control of the kit.

Bone Marrow isolation, culture, and treatment. Bone marrow was extracted from the ischium, femur and tibia into HBSS containing 5% FBS by centrifugation at 8000 rpm for 5 min. The cell pellet was resuspended in sterile 1X RBC lysis buffer for 10 mins followed by washing with 1X MACS buffer. The cells were then resuspended in DMEM containing 10% FBS and 1% Pen/Strep

and counted on a TC20™ automated cell counter (Bio-Rad). 2 x 10^6 cells were seeded in each well of a Costar® ultra-low attachment, flat-bottom, polystyrene, 24 well plate (Corning, cat# 3473) in a total volume of 300 µl. *Treatment with plasma:* Bone marrow cells were treated with plasma that had been collected from PBS-treated (uninfected controls) or UPEC-infected mice at a final concentration of 0.5% (v/v). In total, plasma samples from 8 mice/condition were tested in duplicate across three independent experiments. *Sample preparation and flow cytometry:* After the treatment duration, the cells were collected, washed in 1X PBS and fixed with 2% paraformaldehyde for 15-20 min. The fixed cells were washed and resuspended in 1X MACS buffer and stored at 4°C until flow cytometry evaluation. Prior to acquisition, samples were washed in 1X MACS buffer and transferred to a polystyrene round bottom tube fitted with a 35 µm cell strainer cap (Corning, cat# 352235). Flow cytometry sample acquisition was conducted on the BD LSRFortessa Dual SORP (BD Biosciences) using BD FACSDiva™ v9 software (BD Biosciences). Data was analyzed using FlowJo™ v10 software (BD Life Sciences).

TIMP1 neutralizing antibody treatment. Mice were infected with UPEC and those with urine bacterial titers exceeding 5 x 10^4 CFU/mL, at 48 hours p.i., were separated into 2 treatment groups. Either normal goat IgG control antibody (R&D Systems, cat# AB-108-C, 1 mg/ml) or goat anti-mouse TIMP1 neutralizing antibody (R&D Systems, cat# AF980, 0.2 mg/ml) was diluted in sterile, 1X PBS and administered at a concentration of 32 mg/kg, by intraperitoneal injection, every 48 hours up to 2 weeks p.i, the experimental endpoint[15]. In the nulliparous model, of the 37 animals that received UPEC, 27 were confirmed UTI bearing at 48 hours p.i. and of these 27 UTI-bearing animals, 15 were stratified into the IgG control group and 12 into the TIMP1 treatment group. At endpoint, 7/15 IgG animals remained UTI-bearing while 8/12 TIMP1 animals remained UTI-bearing. In the PLI model, of 20 animals that received UPEC, 16 were confirmed UTI-bearing at 48 hours p.i. and of these 16 UTI-bearing animals, 10 were stratified into the TIMP1 treatment group and 6 into the IgG control group. At endpoint, 2/10 TIMP1 treated animals remained UTI-bearing, while 4/6 IgG treated animals remained UTI-bearing.

CSF3 neutralizing antibody treatment. Mice were infected with UPEC and those with urine bacterial titers exceeding 5 x 10^4 CFU/mL, at 48 hours p.i., were separated into 2 treatment groups. For CSF3 neutralization, mice were administered either rat IgG1 isotype controls (R&D Systems, cat# MAB005) or rat anti-mouse CSF3 neutralization (R&D Systems, cat# MAB414) diluted in sterile PBS at a concentration of 12 ug/mouse in the same course as described above for TIMP1 neutralization[16]. In the nulliparous model, of the 28 animals that received UPEC, 18 were confirmed UTI bearing at 48 hours p.i. and of these 18 UTI-bearing animals, 14 were

stratified into the CSF3 treatment group and 11 into the IgG control group. At endpoint (2 weeks p.i.), 6/11 IgG animals remained UTI-bearing while 5/14 CSF3 animals remained UTI-bearing.

scRNA-seq sample preparation. For all conditions, mammary tissues were collected and digested to a single cell suspension as described above including subjecting the pellet to two rounds of RBC lysis (10 min each) in 1X RBC lysis buffer (BioLegend, cat# 420301). Cell suspensions with a viability greater than 70% were used for scRNA-seq library preparation on the 10X Chromium System (10X Genomics). Single cell libraries were then sequenced (single-end) and indexed using the NextSeq 550 High Output Sequencer (Illumina). Mammary tissue from nulliparous UTI-bearing animas (n=2) yielded a total of 7,100 sequenced cells and post-lactation involution (PLI) control mice (PLI-PBS) (n=2) yielded a total of 11,887 sequenced cells and UTI-bearing (PLI-UTI) (n=2) yielded a total of 9,086 sequenced cells from scRNA-seq. Nulliparous No UTI datasets were previously generated and described[10].

scRNA-seq data processing and analysis. scRNA-seq reads were aligned to the mm10 mouse genome assembly using Cell Ranger v.3.1.0 (10X Genomics). Data pre-processing was carried out using Seurat v.4.1.1 using a previously described method[10,17]. Briefly, cells with fewer than 200 or greater than 6000 genes or those with mitochondrial gene content higher than 25% were excluded. Batch correction was performed on integrated data, using in-built Seurat protocols. Specifically, the FindIntegrationsAnchors() function to identify anchors followed by the IntegrateData() function was used. Principal component analysis (with the top 2000 differentially expressed genes) and unsupervised, hierarchical clustering (resolution = 0.5) was conducted on each Seurat. Gene signature expression scoring was carried out using the AddModuleScore_UCell() function from the UCell package[18]. Statistical analysis on gene expression scores and individual genes was done through stat_compare_means(), a function included in package ggpubr. To quantify cellular cluster population abundance, we utilized Propeller from the Speckle package to quantify statistically significant differences in population abundance between conditions for each cluster[19].

Differential expression between conditions in each cell type was examined through FindMarkers(). This generated gene list was then ranked based on avg_log2FC value and saved as .rnk file for input into Gene Set Enrichment Analysis (GSEA) v4.1 for pathway analysis of Hallmark Terms. Pathways are visualized using ggplot().

Epithelial cell analysis*.* Cell clusters composed of mammary epithelial cells from nulliparous (NP) or post-lactation involution (PLI) mice, were identified by their expression of genes *Epcam*,

Krt8, *Krt18*, *Krt5* and/or *Krt14* and extracted from the datasets, re-clustered and re-assessed for quality. Such approach yielded the following cell numbers: NP UTI-bearing = 3,549 cells; NP no UTI = 1,991 cells; PLI control mice (PBS) = 5,350 cells; and PLI UTI-bearing mice = 2,967 cells. These cells were then classified into the three major epithelial cell lineage populations (BM, LASP, LHS) and utilized for further analysis based on previously defined marker genes[20].

Fibroblast cell analysis. Cell clusters composed of fibroblasts from nulliparous (NP) or post-lactation involution (PLI) mice were identified by the lack of expression of the genes *Epcam* (epithelial), *Pecam1* (endothelial) and *Ptprc* (immune), and by the expression of the gene *Sparc* as previously described[21,22]. Such approach yielded the following cell numbers: NP UTI-bearing = 1,204 cells; NP no UTI = 84 cells; PLI control mice (PBS) = 2,336 ells; and PLI UTI-bearing mice = 3,607 cells. These cells were then classified into 3 major cellular states according the expression of previously defined maker genes[10].

CellChat analysis. Epithelial clusters (BM, LHS, and LASP) and fibroblast clusters (F1, F2, F3) identified above were merged in order to facilitate examination of changes to cellular signaling between epithelial and fibroblast populations using CellChat[23]. In brief, populations were subsetted based on condition (nulliparous: No-UTI, UTI; post-lactation involution: PBS, UTI) and CellChat objects were generated for each condition in each dataset the pre-processed according to previous literature[23]. Visualization of cell signaling networks were generated through functions provided by the CellChat package such as netAnalysis_signalingRole_heatmap() and netVisual_bubble().

Gene signatures analysis summary. The analysis of epithelial lineage identification was performed as previously described[10]. The analysis of YAP signaling signature was performed with a previously described gene list [24]. The analysis of ECM signaling signature was performed with a previously described gene list (KEGG pathway analysis– source hsa04512). The analysis of ECM signaling signature was performed with a previously described gene list (GSEA pathway analysis– source WP4534). The analysis of Fibroblast Activation signature was performed with a previously described gene list[25]. The analysis of Neutrophil recruitment signature was performed with a previously described gene list[26].

Statistics and Reproducibility. Data represents results from at least three independent experiments. All statistical analyses were conducted using GraphPad Prism 7. For tissue analysis, a linear mixed-effects model was utilized to analyze the percent area data, with groups serving as the fixed effect, and random effects incorporated to account for the correlation between the

outcome variable within the same mouse nested within the same images. To investigate differences in percent area, simultaneous tests for general linear hypotheses of contrasts of interest were conducted. To adjust for multiple comparisons, p-values were corrected using the Tukey's method. A linear mixed-effects model was also utilized to analyze the log-transformed percent data (to adjust for normality), with groups serving as the fixed effect, and random effects incorporated to account for the correlation between the outcome variable within the same mouse nested within the same images. To investigate differences in log-transformed percent data, simultaneous tests for general linear hypotheses of contrasts of interest were conducted. To adjust for multiple comparisons, p-values were corrected using the Tukey's method. For comparison of two conditions, an unpaired t-test with Welch's correction was conducted, while for comparisons of >2 conditions, one-way ANOVA with Tukey's multiple corrections was used. Adjusted p-values lower than 0.05 were considered statistically significant. For gene expression quantification, statistical analysis was conducted using a non-parametric Wilcox test between 2 groups of samples. Across the manuscript *$p \leq 0.05$; **$p \leq 0.01$; ***$p \leq 0.001$; ****$p \leq 0.0001$.

Statistical analysis was supported by the CSHL Biostatistician, Taehoon Ha.

Supplementary Figures

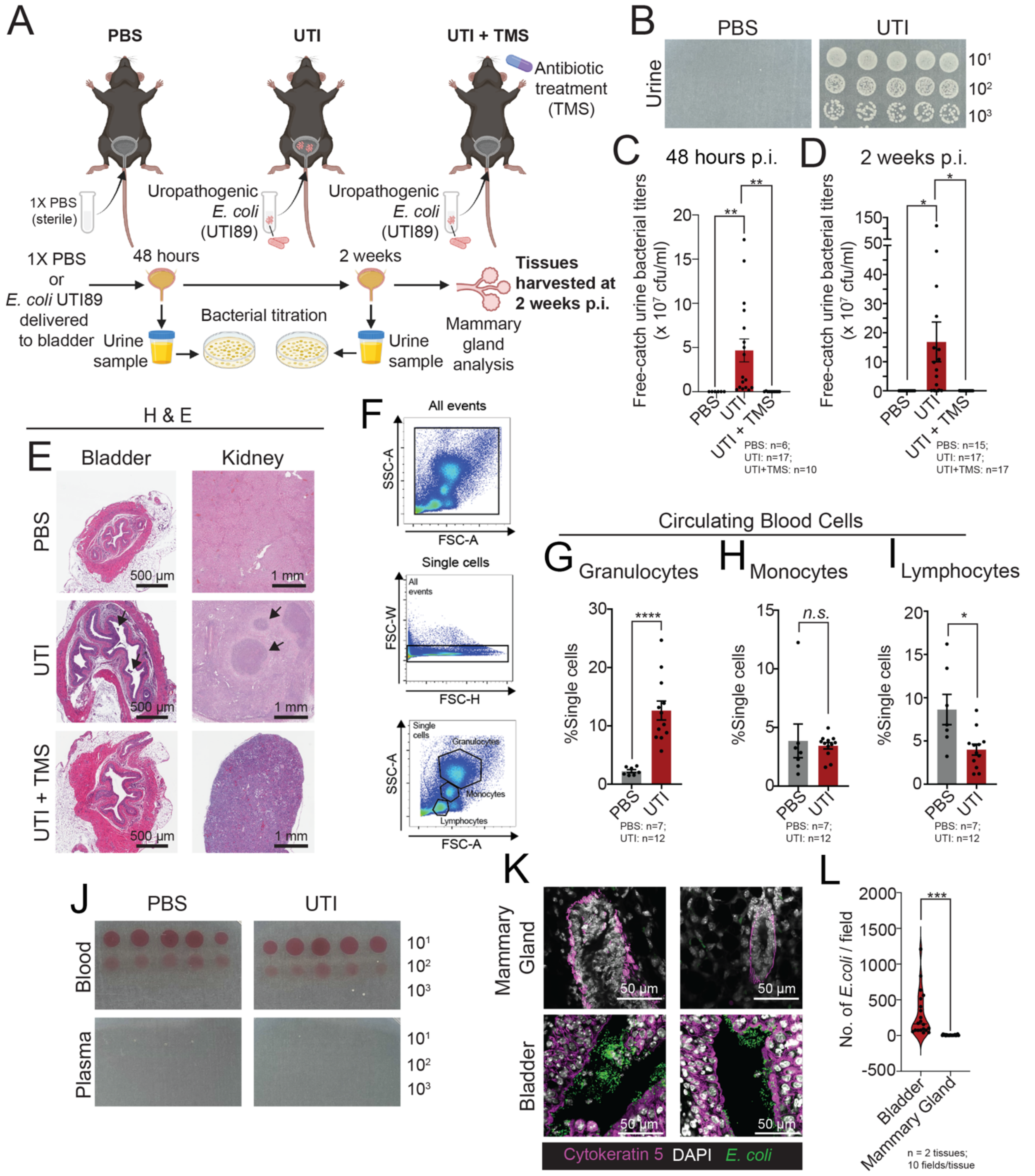

Supplementary Figure 1 (related to Main Figure 1). UPEC infection in nulliparous C57BL/6 mice. (A) Experimental design showing nulliparous administered with either 1X PBS (PBS),

uropathogenic *E. coli* strain UTI89 (UTI) transurethrally. A subset of *E. coli* infected mice was also treated with antibiotic TMS treatment (UTI+TMS). Bacterial titers of free-catch urine samples were conducted at 48 hours post-infection and 2 weeks post-infection (p.i.). Tissues were assessed at 2 weeks p.i. Created with BioRender.com. **(B)** Example of LB agar spot cultures of urine obtained from PBS control mice, or UTI-bearing mice. Diluted 10-fold from 10^{-1} to 10^{-3} in sterile 1X PBS. **(C-D)** Bacterial titers of free-catch urine samples, collected at (**C**) 48 hours ,**p = 0.0024 (PBS vs UTI) and **p = 0.0025 (UTI+TMS vs UTI), and (**D**) 2 weeks post-infection (p.i.) from PBS control mice, UTI-bearing mice, and from UTI-bearing mice treated with TMS. Error bars represent mean ± S.E.M. Statistical analysis was conducted with an unpaired t-test with Welch's correction. *$p \leq 0.05$; UTI; *p = 0.0172 (PBS vs UTI) and *p = 0.0135 (UTI+TMS vs UTI). (**E**) H&E-staining in histological sections of bladder and kidney tissues from PBS control mice and UTI-bearing mice. Representative images shown from n=15 PBS, n=17 UTI, n=15 UTI+TMS. Scale bar = 500 μm and 1 mm, respectively. Arrows indicate signs of urothelial erosion and immune infiltration as confirmed by pathological analysis. **(F-I)** Flow cytometry quantification gating strategy of blood cells at 2 weeks p.i. of **(G)** granulocytes (****p < 0.0001), **(H)** monocytes (p = 0.783) and **(I)** lymphocytes (*p = 0.0387) in blood collected from PBS control mice (n=7) and UTI-bearing mice (n=12). Error bars represent mean ± S.E.M. Statistical analysis was conducted with an unpaired t-test with Welch's correction. **(J)** LB agar spot cultures of blood and plasma collected from PBS control mice and UTI-bearing mice at 2 weeks p.i. Diluted 10-fold from 10^{-1} to 10^{-3} in sterile PBS (representative images shown from n=5 PBS, n=5 UTI). **(K)** Immunofluorescence staining of cytokeratin 5 (magenta), DAPI (white) and *E. coli* (green) in mammary gland and bladder tissue sections from UTI-bearing mice. Scale bar = 50 μm. n=2 tissues. **(L)** *E. coli* staining quantification in mammary gland and bladder tissue sections from UTI-bearing mice. *E. coli* counted for 10 fields per tissue section. Violin plots show data distribution with median (solid line) and quartiles (dashed line) indicated. Statistical analysis was conducted with an unpaired t-test with Welch's correction; ***p = 0.0006. Source data and p-values are provided as a Source Data file.

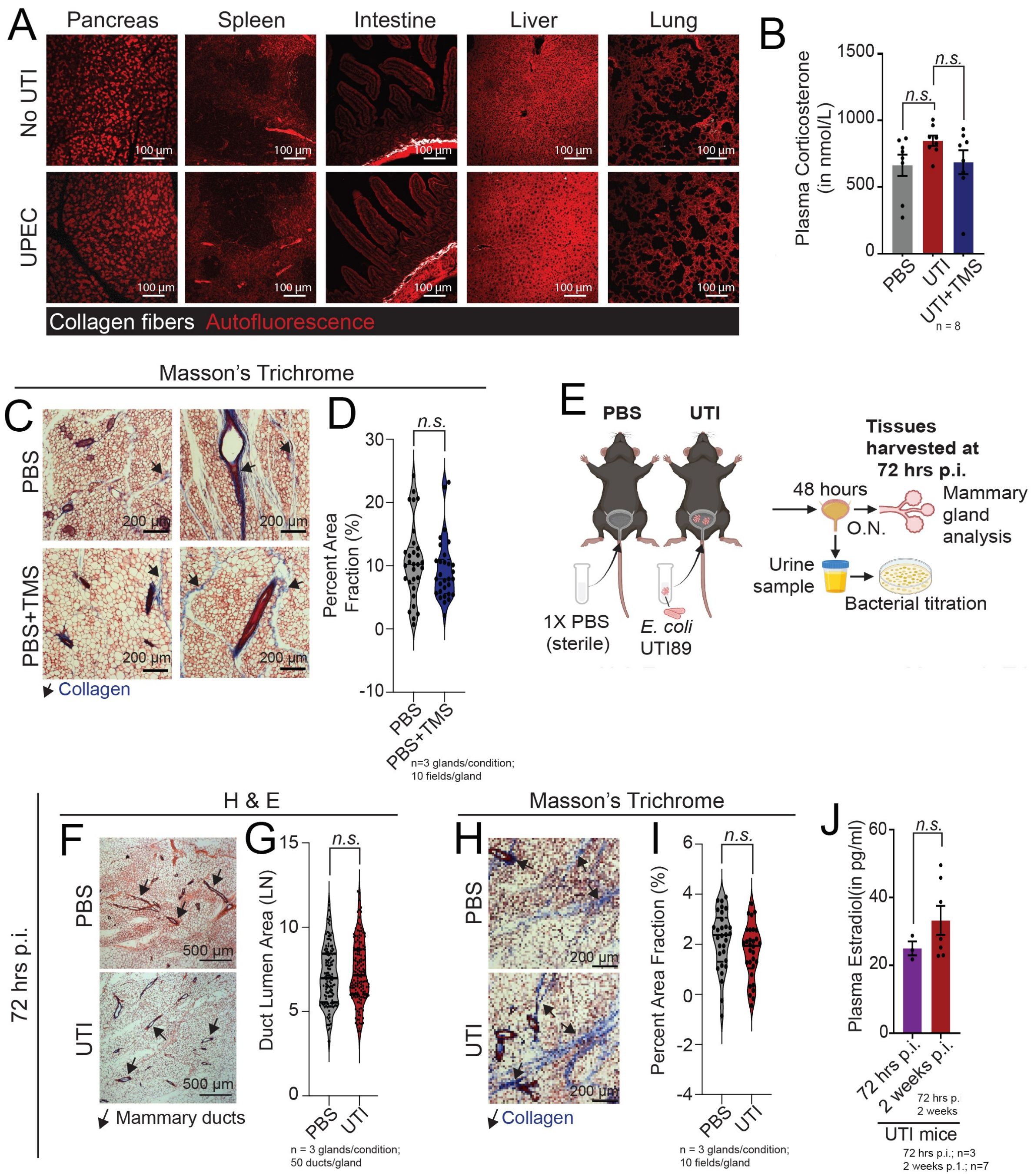

Supplementary Figure 2 (Related to Main Figure 1). Characterization of ECM alterations in nulliparous UTI-bearing mice. (A) Two photon microscopy and second harmonic generation images of H&E-stained tissue sections from pancreas, spleen, intestine, liver and lungs of PBS

control mice (n=1) and UTI-bearing mice (n=2), showing collagen fibers (white) and tissue auto-florescence (red). Scale bar = 100 μm. (**B**) Elisa quantification of plasma corticosterone levels in PBS control mice (n=8), UTI-bearing mice (n=8), and UTI-bearing mice treated with TMS (n=8) at 2 weeks post infection (p.i.). Error bars represent mean ± S.E.M. Statistical analysis was conducted with one-way ANOVA with Tukey's multiple correction; p = 0.1954 (PBS vs UTI) and p = 0.2838 (UTI+TMS vs UTI) (**C-D**) Masson Trichrome blue staining of mammary tissue sections and **(D)** quantification of positively stained collagen (in blue) from PBS control mice (n=3), PBS control mice treated with TMS (2 weeks of treatment, n=3). Scale bar = 200 μm. Arrows indicate positively stained collagen (in blue). Violin plots show data distribution with median (solid line) and quartiles (dashed line) indicated. Statistical analysis was conducted with an unpaired t-test with Welch's correction; p = 0.3422. **(E)** Experimental design showing nulliparous mice administered 1X PBS (PBS) or uropathogenic *E. coli* strain UTI89 (UTI). Bacterial titers of free-catch urine samples were conducted at 48 hours p.i. and tissues were assessed at 72 hours p.i. Created with BioRender.com. **(F-G)** H&E staining of mammary tissue sections and **(G)** quantification of ductal lumen area of mammary tissue sections from PBS control mice (n=3) and UTI-bearing mice (n=3) at 72 hours p.i. Scale bar = 500 μm. Arrows indicate mammary ducts. Area measured for 50 ducts/gland. Violin plots show data distribution with median (solid line) and quartiles (dashed line) indicated. Statistical analysis was conducted with an unpaired t-test with Welch's correction; p = 0.2669. **(H-I)** Masson's trichrome staining of mammary tissue sections and **(I)** quantification of positively stained collagen (blue) from PBS control mice (n=3) and UTI-bearing mice (n=3), at 72 hours p.i. Scale bar = 200 μm. Arrows indicate areas of positively stained collagen (in blue). Area measured for 10 fields/gland. Violin plots show data distribution with median (solid line) and quartiles (dashed line) indicated. Statistical analysis was conducted with an unpaired t-test with Welch's correction; p = 0.1192. (**J**) Elisa quantification of plasma estradiol levels in UTI-bearing mice at 72 hours (n=3), and 2 weeks (n=7) p.i. Error bars represent mean ± S.E.M. Statistical analysis was conducted with an unpaired t-test with Welch's correction; p = 0.1192. Source data and p-values are provided as a Source Data file.

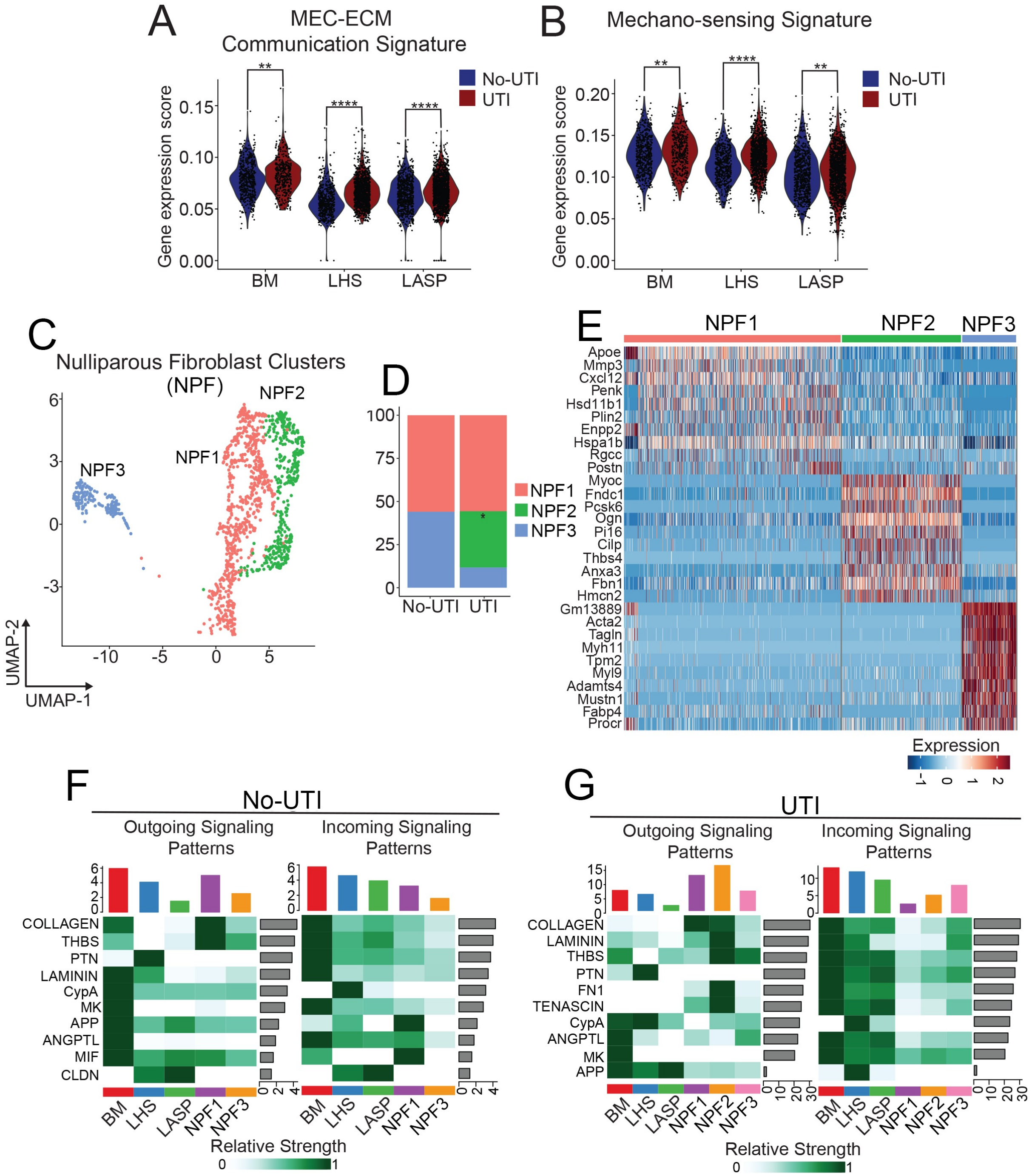

Supplementary Figure 3 (Related to Main Figure 2). scRNA-seq analysis of mammary tissue from uninfected and UTI-bearing mice. (A-B) Violin plot showing gene expression scores of **(A)** MEC-ECM Communication signatures (BM: **p = 0.0015, LHS: ****p < 2.22e-16, LASP: ****p =

2.30E-06) and **(B)** Mechano-sensing signatures (BM: **p = 0.0064, LHS: ****p < 2.22e-16, LASP: **p = 0.0015) in MECs from uninfected mice (No-UTI, blue, n=2) and UTI-bearing mice (red, n=2). Statistical analysis was conducted with a non-parametric Wilcox test. (**C-D**) Fibroblast (**C**) UMAP clustering and (**D**) abundance quantification in mammary tissue of uninfected mice (No-UTI) and UTI-bearing mice. Statistical analysis was carried out with Propeller pipeline[19]; *p = 0.011988. (**E**) Heatmap showing top 10 differentially expressed genes across populations of mammary fibroblasts. (**F-G**) CellChat heatmaps showing pathways inferred to be enriched either as outgoing or incoming signaling patterns in MECs (BM, LHS, LASP) or fibroblasts (NF1, NF2, NF3) from uninfected mice (No-UTI) and UTI-bearing mice. Colored bar plots indicate communication signal intensity. Gray side bar plots indicate enrichment for specific signaling pathways.

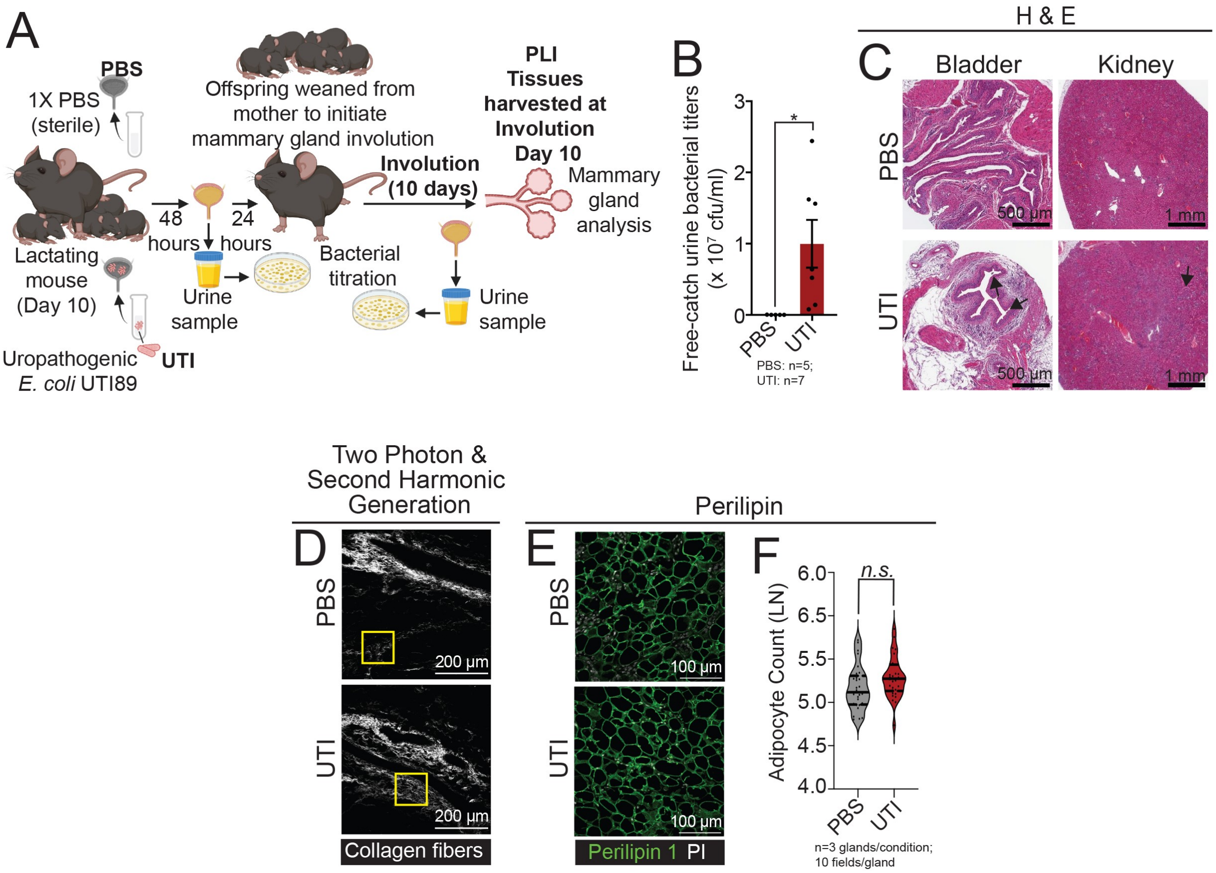

Supplementary Figure 4 (Related to Main Figure 3). UTI infection of post-lactation involuting female mice. (A) Experimental design showing the utilization of post-lactation involuting female mice (PLI). On lactation day 10, the mice were administered 1X PBS (PBS) or uropathogenic *E. coli* strain UTI89 (UTI) transurethrally. Urine bacterial titers were conducted at 48 hours p.i. and pups were weaned the following day to allow for forced mammary gland involution. Urine bacterial titers and tissue analyses were conducted at forced involution day 10. Created with BioRender.com. **(B)** Bacterial titers of free-catch urine samples from PBS control PLI mice (n=5) and UTI-bearing PLI mice (n=7). Error bars represent mean ± S.E.M. Statistical analysis was conducted with an unpaired t-test with Welch's correction; *p = 0.0246. (**C**) H&E staining in histological sections of bladder and kidney tissues from PBS control and UTI-bearing PLI mice. Scale bar = 500 µm and 1 mm, respectively. Arrows indicate signs of urothelial erosion and immune infiltration as confirmed by pathological analysis. (**D**) Two photon microscopy and second harmonic generation of H&E-stained mammary tissue sections from PBS control and UTI-bearing PLI mice. Yellow square indicates areas with positive collagen signal (white). (**E-F**) Immunostaining of mammary tissue sections and (**F**) quantification of Perilipin+ adipocytes (green), PI (white), in PBS control (n=3) and UTI-bearing PLI mice (n=3). Scale bar = 100 µm.

Violin plots show data distribution with median (solid line) and quartiles (dashed line) indicated. Statistical analysis was conducted with an unpaired t-test with Welch's correction; $p = 0.0689$. Source data and p-values are provided as a Source Data file.

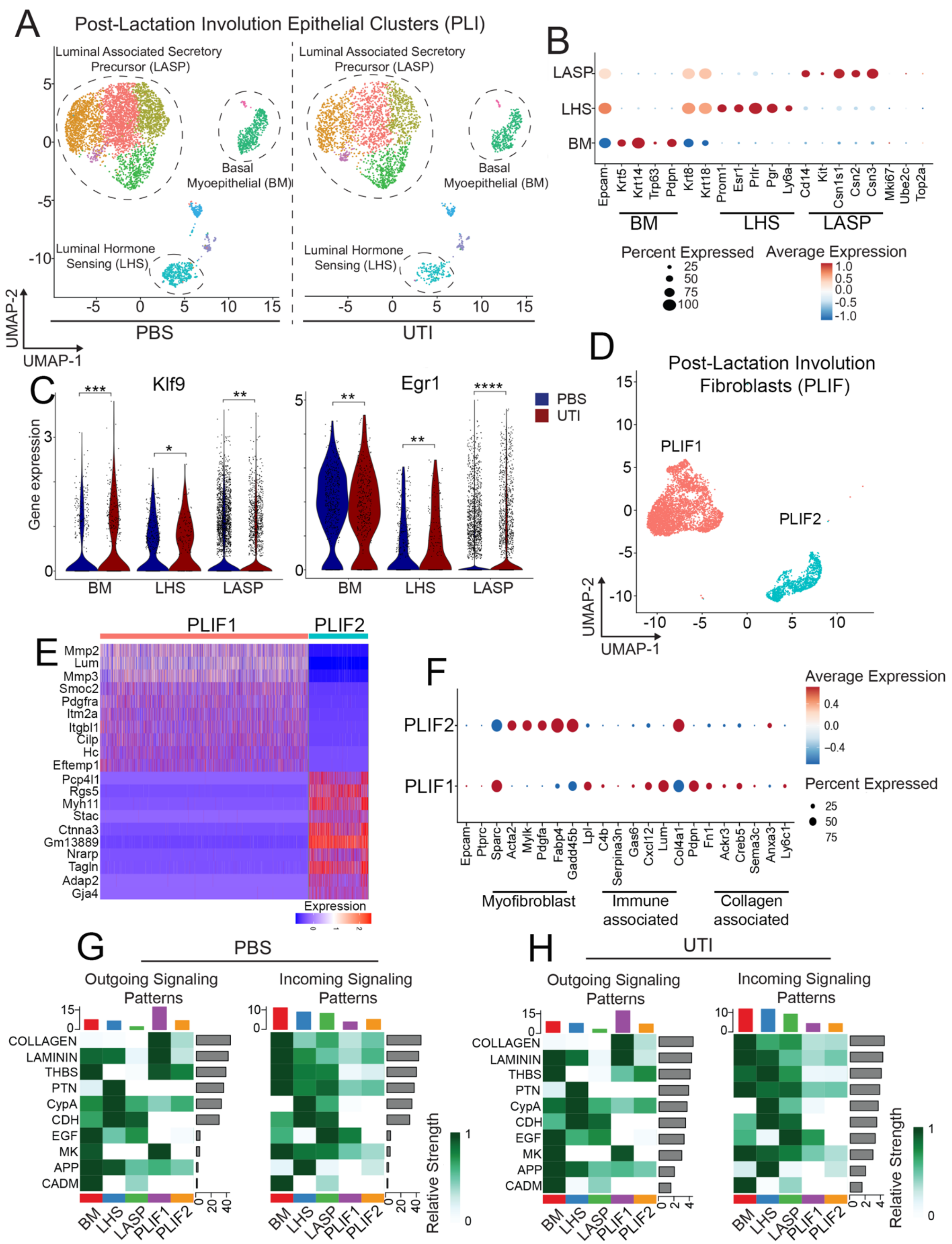
A
Post-Lactation Involution Epithelial Clusters (PLI)
Luminal Associated Secretory Precursor (LASP)
Basal Myoepithelial (BM)
Luminal Hormone Sensing (LHS)
PBS
UTI
UMAP-1
UMAP-2
B
LASP
LHS
BM
Epcam Krt5 Krt14 Trp63 Pdpn Krt8 Krt18 Prom1 Esr1 Prlr Pgr Ly6a Cd14 Kit Csn1s1 Csn2 Csn3 Mki67 Ube2c Top2a
Percent Expressed
Average Expression
C
Klf9
Egr1
Gene expression
PBS
UTI
D
Post-Lactation Involution Fibroblasts (PLIF)
PLIF1
PLIF2
E
Mmp2 Lum Mmp3 Smoc2 Pdgfra Itm2a Itgbl1 Cilp Hc Eftemp1 Pcp4l1 Rgs5 Myh11 Stac Ctnna3 Gm13889 Nrarp Tagln Adap2 Gja4
Expression
F
Epcam Ptprc Sparc Acta2 Mylk Pdgfa Fabp4 Gadd45b Lpl C4b Serpina3n Gas6 Cxcl12 Lum Col4a1 Pdpn Fn1 Ackr3 Creb5 Sema3c Anxa3 Ly6c1
Myofibroblast
Immune associated
Collagen associated
Average Expression
Percent Expressed
G
PBS
H
UTI
Outgoing Signaling Patterns
Incoming Signaling Patterns
COLLAGEN LAMININ THBS PTN CypA CDH EGF MK APP CADM
BM LHS LASP PLIF1 PLIF2
Relative Strength

Supplementary Figure 5 (Related to Main Figure 3). scRNA-seq analysis of mammary tissue from control and UTI-bearing PLI mice. (A-B) UMAP and (**B**) lineage classification of mammary epithelial cells from PBS control (n=2) and UTI-bearing PLI mice (n=2). (**C**) Violin plot showing *Egr1* and *Klf9* mRNA levels across MEC lineages from control and UTI-bearing PLI mice. Statistical analysis was conducted with a non-parametric Wilcox test; Klf9: BM: ***p = 0.0016, LHS: *p = 0.048, **p = 0.0095; Egr1: BM: **p = 0.0026, LHS: **p = 0.0035, LASP: ****p = 2.50e-07. (**D**) Fibroblast UMAP clustering. (**E**) Heatmap showing top 10 differentially expressed genes across populations of mammary fibroblasts. (**F**) Dot plot showing average expression of genes of myofibroblast state, and those of Immune-associated and collage-associated fibroblasts. (**G-H**) CellChat heatmaps showing pathways inferred to be enriched either as outgoing or incoming signaling patterns in MECs (BM, LHS, LASP) or fibroblasts (PLIF1, PLIF2) PBS control and UTI-bearing PLI mice. Colored bar plots indicate communication signal intensity. Gray side bar plots indicate enrichment for specific signaling pathways.

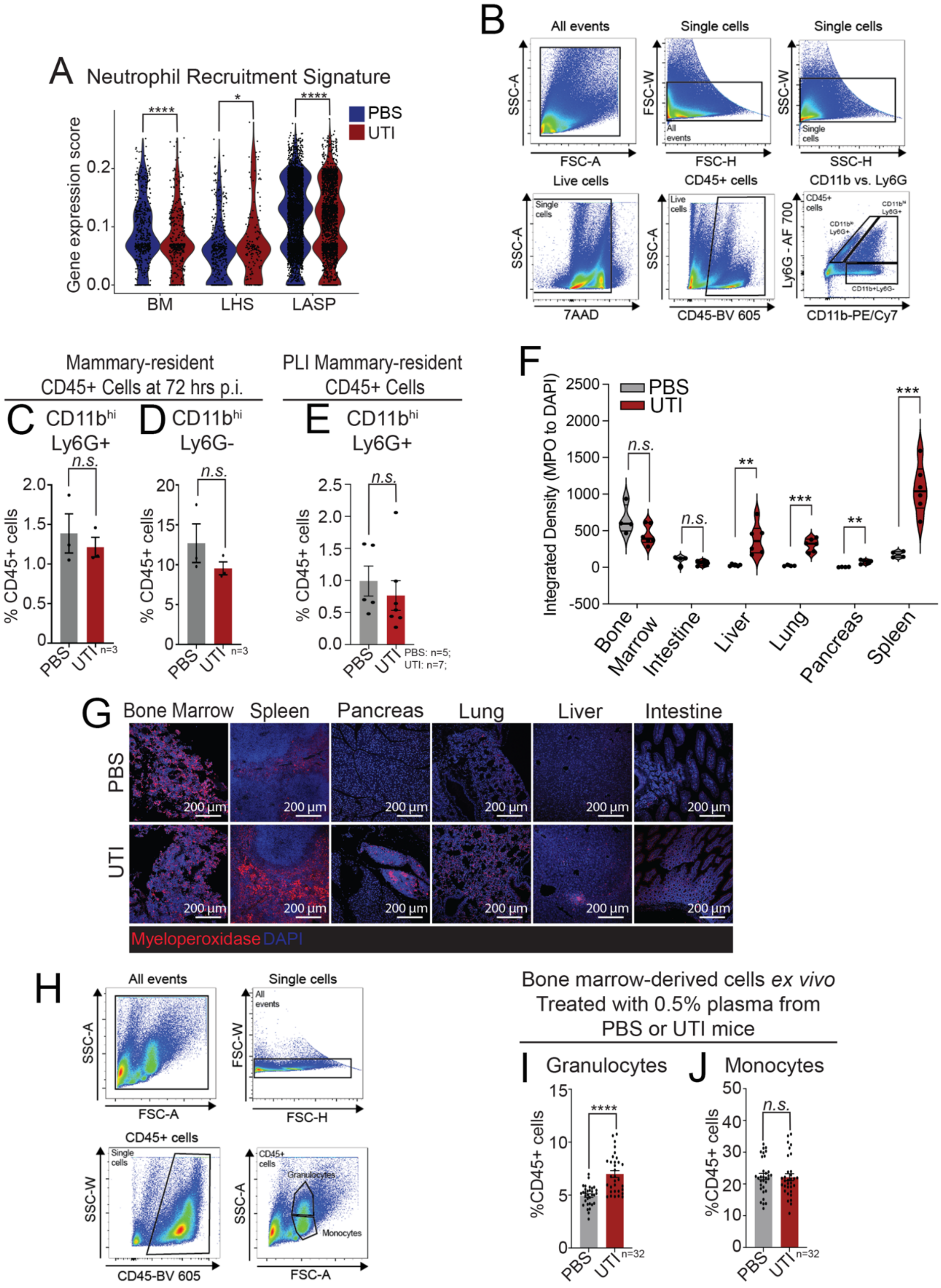
A Neutrophil Recruitment Signature
PBS
UTI
Gene expression score
BM
LHS
LASP
B
All events
Single cells
Single cells
SSC-A
FSC-A
FSC-W
FSC-H
SSC-W
SSC-H
Live cells
CD45+ cells
CD11b vs. Ly6G
7AAD
CD45-BV 605
Ly6G - AF 700
CD11b-PE/Cy7
Mammary-resident CD45+ Cells at 72 hrs p.i.
PLI Mammary-resident CD45+ Cells
C CD11bhi Ly6G+
D CD11bhi Ly6G-
E CD11bhi Ly6G+
% CD45+ cells
n.s.
n=3
PBS: n=5; UTI: n=7;
F
Integrated Density (MPO to DAPI)
Bone Marrow
Intestine
Liver
Lung
Pancreas
Spleen
G
Bone Marrow
Spleen
Pancreas
Lung
Liver
Intestine
200 µm
Myeloperoxidase DAPI
H
All events
Single cells
CD45+ cells
Granulocytes
Monocytes
Bone marrow-derived cells ex vivo Treated with 0.5% plasma from PBS or UTI mice
I Granulocytes
J Monocytes
%CD45+ cells
n=32

Supplementary Figure 6 (Related to Main Figure 4). Mammary immune alterations in UTI-bearing mice. (A) Violin plot showing gene expression scores of Neutrophil recruitment signature in MECs from control mice (PBS) and UTI-bearing mice. Statistical analysis was conducted with a non-parametric Wilcox test; BM: ****p = 5.00e-05, LHS: *p = 0.03, LASP: ****p = 7.40e-10. **(B)** Flow cytometry gating strategy utilized for the analysis of mammary localized neutrophils. **(C-D)** Flow cytometry analysis of neutrophil populations in the mammary tissue of nulliparous PBS control mice (n=3) and UTI-bearing mice (n=3) 72 hours p.i. Error bars represent mean ± S.E.M. Statistical analysis was conducted with a non-parametric Wilcox test; CD11b+ Ly6G+: p = 0.5766, CD11b+ Ly6G-: p = 0.3230. **(E)** Flow cytometry analysis of neutrophil $CD11b^{+}Ly6G^{+}$ cells in the mammary tissue of PBS control mice (n=5) and UTI-bearing mice (n=7) PLI mice, 2 weeks p.i. Error bars represent mean ± S.E.M. Statistical analysis was conducted with a non-parametric Wilcox test; p = 0.5095. **(F-G)** Immunostaining of mammary tissue sections and quantification of Myeloperoxidase+ neutrophils (Mpo+, red) in the bone marrow, spleen, pancreas, lung, liver and intestine of nulliparous PBS control mice and UTI-bearing mice, 2 weeks p.i. Representative images shown from n=6 UTI and n=6 PBS for liver, n=6 UTI and n=4 PBS for the bone marrow, intestine, liver, lung, pancreas, and spleen. Scale bar = 200 µm. Violin plots show data distribution with median (solid line) and quartiles (dashed line) indicated. Statistical analysis was conducted with a nonparametric Wilcox test; Bone Marrow: p = 0.087742, Intestine: p =0.198302, Liver: **p = 0.002225, Lung: ***p = 0.000215, Pancreas: **p = 0.001077, Spleen: ***p = .000781. **(H)** Flow cytometry gating strategy utilized for the analysis of bone marrow cultured cells **(I-J)** Flow cytometry analysis of granulocytes (****p < 0.0001) and monocytes (p = 0.8688) in *ex vivo* cultured bone marrow cells after treatment with 0.5% (v/v) plasma from PBS control mice (n=8) or UTI-bearing mice (n=8) for 24 hours. n=32/condition. Error bars represent mean ± S.E.M. Statistical analysis was conducted with a non-parametric Wilcox test. Source data and p-values are provided as a Source Data file.

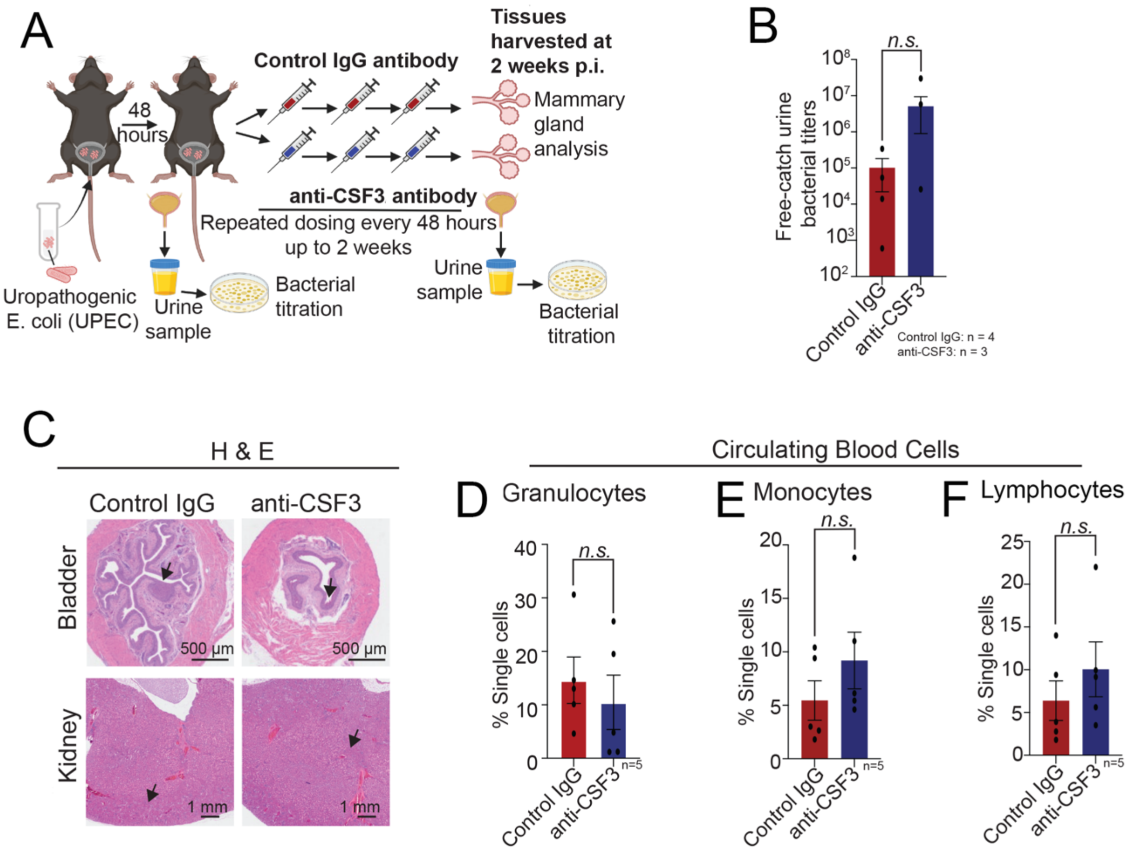

Supplementary Figure 7 (Related to Main Figure 4). Mammary immune alterations in UTI-bearing mice. (A) Experimental design showing approach to treat UTI-bearing mice with IgG control or anti-CSF3 neutralizing antibody (2 weeks, 6 doses). Urine bacterial titers were conducted at 48 hours p.i. Urine bacterial titers and tissue analyses were conducted 24 hours after the last treatment dose. Created with BioRender.com. (**B**) Bacterial titers of free-catch urine samples from IgG treated (n=4) or anti-CSF-3 neutralizing antibody treated (n=3) UTI-bearing mice. Error bars represent mean ± S.E.M. Statistical analysis was conducted with a non-parametric Wilcox test; p = 0.2802. (**C**) H&E-staining in histological sections of bladder and kidney tissues from IgG treated or anti-CSF3 neutralizing antibody treated, UTI-bearing mice. Representative images shown from n=4 control IgG, n=3 anti-CSF3. Scale bar = 500 µm and 1 mm, respectively. Arrows indicate signs of urothelial erosion and immune infiltration as confirmed by pathological analysis. **(D-F)** Flow cytometry quantification of **(D)** granulocytes (p = 0.5549), **(E)**

monocytes (p = 0.2821) and **(F)** lymphocytes (p = 0.3844) in blood collected from IgG treated (n=5) or anti-CSF3 neutralizing antibody treated (n=5), UTI-bearing mice. Error bars represent mean ± S.E.M. Statistical analysis was conducted with a non-parametric Wilcox test. Source data and p-values are provided as a Source Data file.

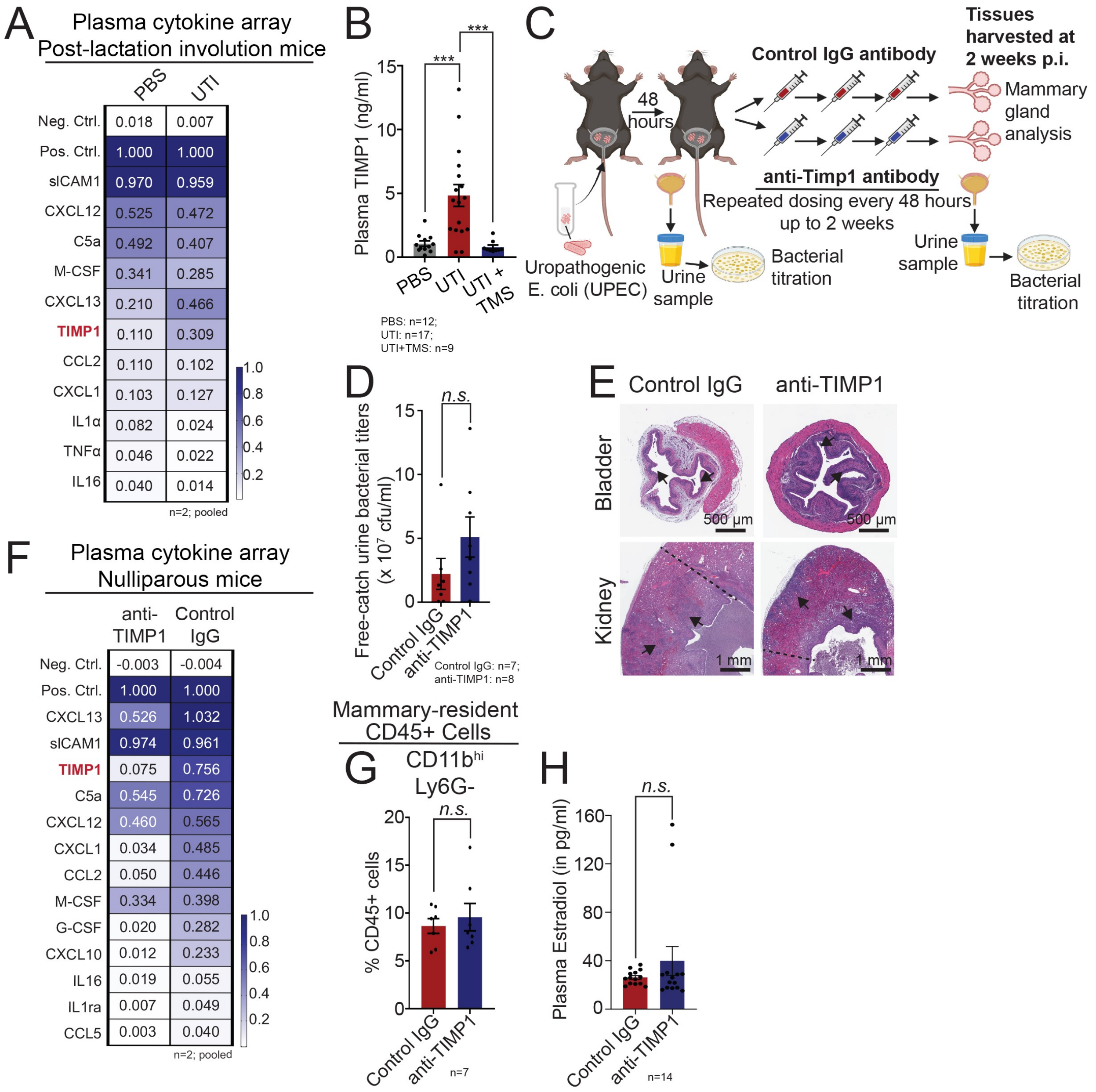

Supplementary Figure 8 (Related to Main Figure 5). Treatment of nulliparous UTI-bearing mice with TIMP1 neutralizing antibodies. (**A**) Cytokine analysis of plasma from PBS control and UTI-bearing post-lactation involution (PLI) mice. (**B**) Elisa quantification of plasma TIMP1 levels in PBS control mice (n=12), UTI-bearing mice (n=17), and UTI-bearing mice treated with TMS (n=9). Error bars represent mean ± S.E.M. Statistical analysis was conducted with one-way ANOVA with Tukey's multiple correction; PBS vs UTI: ***p = 0.0007, UTI+TMS vs UTI: ***p = 0.0008. (**C**) Experimental design showing approach to treat UTI-bearing mice with IgG control or anti-TIMP1 neutralizing antibody (2 weeks, 6 doses). Urine bacterial titers were conducted at 48

hours p.i. Urine bacterial titers and tissue analyses were conducted 24 hours after the last treatment. Created with BioRender.com. (**D**) Bacterial titers of free-catch urine samples from IgG treated (n=7) or anti-TIMP1 neutralizing antibody treated (n=8) UTI-bearing mice. Error bars represent mean ± S.E.M. Statistical analysis was conducted with a non-parametric Wilcox test; p = 0.1698. (**E**) H&E-staining in histological sections of bladder and kidney tissues from IgG treated or anti-TIMP1 neutralizing antibody treated, UTI-bearing mice. Representative images shown from n=7 control IgG, n=8 anti-TIMP1. Scale bar = 500 μm and 1 mm, respectively. Arrows indicate signs of urothelial erosion and immune infiltration as confirmed by pathological analysis. (**F**) Cytokine analysis of plasma from IgG treated or anti-TIMP1 neutralizing antibody treated UTI-bearing nulliparous mice. (**G**) Flow cytometry analysis of CD11b+Ly6G- mammary myeloid cells from IgG treated or anti-TIMP1 neutralizing antibody treated, UTI-bearing mice. Error bars represent mean ± S.E.M. Statistical analysis was conducted with a non-parametric Wilcox test; p = 0.5845. (**H**) Elisa (ELISA) quantification of plasma estradiol levels in IgG treated (n=14) or anti-TIMP1 neutralizing antibody treated (n=14) UTI-bearing nulliparous mice. Error bars represent mean ± S.E.M. Statistical analysis was conducted with a non-parametric Wilcox test; p = 0.2715. Source data and p-values are provided as a Source Data file.

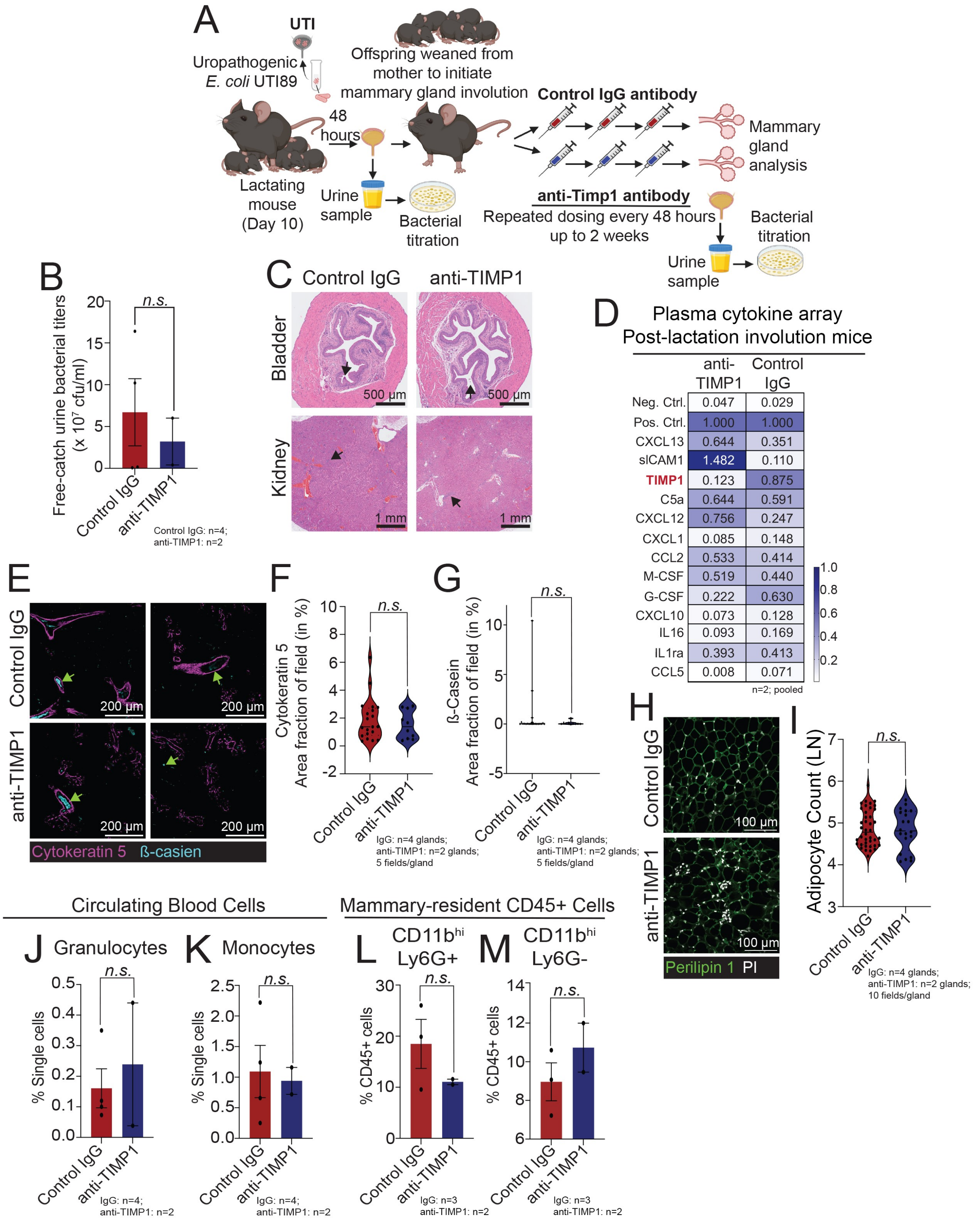

Supplementary Figure 9 (Related to Main Figure 5). Treatment of post-lactating involuting (PLI) UTI-bearing mice with TIMP1 neutralizing antibodies. (**A**) Experimental design showing

approach to treat UTI-bearing PLI mice with IgG control or anti-TIMP1 neutralizing antibody (2 weeks, 6 doses). Urine bacterial titers were conducted at 48 hours p.i. Urine bacterial titers and tissue analyses were conducted 24 hours after the last treatment. Created with BioRender.com. (**B**) Bacterial titers of free-catch urine samples from IgG treated (n=4) or anti-TIMP1 neutralizing antibody treated (n=2) UTI-bearing mice. Error bars represent mean ± S.E.M. Statistical analysis was conducted with a non-parametric Wilcox test; p = 0.4982. (**C**) H&E-staining in histological sections of bladder and kidney tissues from IgG treated or anti-TIMP1 neutralizing antibody treated, UTI-bearing mice. Representative images shown from n=4 control IgG, n=2 anti-TIMP1. Scale bar = 500 μm and 1 mm, respectively. Arrows indicate signs of urothelial erosion and immune infiltration as confirmed by pathological analysis. (**D**) Cytokine analysis of plasma from IgG treated or anti-TIMP1 neutralizing antibody treated UTI-bearing PLI mice. (**E-G**) Immunostaining of mammary tissue sections and (**F**) quantification of Cytokeratin 5 (magenta, p = 0.4306) and (**G**) b-casein levels (cyan, p = 0.2449) from UTI-bearing PLI mice after 6 doses of IgG control (n=4) or anti-TIMP1 neutralizing antibody (n=2). Scale bar = 200 μm. Violin plots show data distribution with median (solid line) and quartiles (dashed line) indicated. Statistical analysis was conducted with a non-parametric Wilcox test. (**H-I**) Immunostaining of mammary tissue sections and (**I**) quantification of Perilipin+ adipocytes (green, p = 0.7531) from UTI-bearing PLI mice after 6 doses of IgG control (n=4) or anti-TIMP1 neutralizing antibody (n=2). Scale bar = 100 μm. Violin plots show data distribution with median (solid line) and quartiles (dashed line) indicated. Statistical analysis was conducted with a non-parametric Wilcox test. (**J-K**) Flow cytometry analysis of circulating (**J**) granulocytes (p = 0.7651) and (**K**) monocytes (p = 0.7670) from IgG treated (n=4) or anti-TIMP1 neutralizing antibody treated (n=2), UTI-bearing PLI mice. Error bars represent mean ± S.E.M. Statistical analysis was conducted with a nonparametric Wilcox test. (**L-M**) Flow cytometry analysis of mammary neutrophil populations from IgG treated (n=3) or anti-TIMP1 neutralizing antibody treated (n=2), UTI-bearing PLI mice. Error bars represent mean ± S.E.M. Statistical analysis was conducted with a non-parametric Wilcox test; CD11b+ Ly6G+: p = 0.2604, CD11b+ Ly6G-: p = 0.368. Source data and p-values are provided as a Source Data file.

Supplementary References.

1 O'Brien J, Lyons T, Monks J, Lucia MS, Wilson RS, Hines L, *et al.* Alternatively activated macrophages and collagen remodeling characterize the postpartum involuting mammary gland across species. *Am J Pathol* 2010;**176**:1241–55. https://doi.org/10.2353/ajpath.2010.090735.

2 Lyons TR, O'Brien J, Borges VF, Conklin MW, Keely PJ, Eliceiri KW, *et al.* Postpartum mammary gland involution drives progression of ductal carcinoma in situ through collagen and COX-2. *Nat Med* 2011;**17**:1109–16. https://doi.org/10.1038/nm.2416.

3 Schindelin J, Arganda-Carreras I, Frise E, Kaynig V, Longair M, Pietzsch T, *et al.* Fiji: An open-source platform for biological-image analysis. *Nat Methods* 2012;**9**:676–82. https://doi.org/10.1038/nmeth.2019.

4 Liu Y, Keikhosravi A, Mehta GS, Drifka CR, Eliceiri KW. *Methods for Quantifying Fibrillar Collagen Alignment*. 2017.

5 Lloyd-Lewis B, Sargeant TJ, Kreuzaler PA, Resemann HK, Pensa S, Watson CJ. Analysis of the involuting mouse mammary gland: An in vivo model for cell death. *Methods Mol Biol* 2017;**1501**:165–86. https://doi.org/10.1007/978-1-4939-6475-8_7.

6 Monks J, Orlicky DJ, Libby AE, Dzieciatkowska M, Ladinsky MS, McManaman JL. Perilipin2 promotes lipid droplet-plasma membrane interactions that facilitate apocrine lipid secretion in secretory epithelial cells of the mouse mammary gland. *Front Cell Dev Biol* 2022;**10**:1–17. https://doi.org/10.3389/fcell.2022.958566.

7 Wormsbaecher C, Hindman AR, Avendano A, Cortes-Medina M, Jones CE, Bushman A, *et al.* In utero estrogenic endocrine disruption alters the stroma to increase extracellular matrix density and mammary gland stiffness. *Breast Cancer Res* 2020;**22**:1–12. https://doi.org/10.1186/s13058-020-01275-w.

8 Feigman MJ, Moss MA, Chen C, Cyrill SL, Ciccone MF, Trousdell MC, *et al.* Pregnancy reprograms the epigenome of mammary epithelial cells and blocks the development of premalignant lesions. *Nat Commun* 2020;**11**:1–12. https://doi.org/10.1038/s41467-02016479-z.

9 dos Santos CO, Dolzhenko E, Hodges E, Smith AD, Hannon GJ. An Epigenetic Memory of Pregnancy in the Mouse Mammary Gland. *Cell Rep* 2015;**11**:1102–9. https://doi.org/10.1016/j.celrep.2015.04.015.

10 Henry S, Trousdell MC, Cyrill SL, Zhao Y, Feigman MJ, Bouhuis JM, *et al.* Characterization of Gene Expression Signatures for the Identification of Cellular Heterogeneity in the Developing Mammary Gland. *J Mammary Gland Biol Neoplasia* 2021;**26**:43–66. https://doi.org/10.1007/s10911-021-09486-3.

11 Hanasoge Somasundara AV, Moss MA, Feigman MJ, Chen C, Cyrill SL, Ciccone MF, *et al.* Parity-induced changes to mammary epithelial cells control NKT cell expansion and mammary oncogenesis. *Cell Rep* 2021;**37**:110099. https://doi.org/10.1016/j.celrep.2021.110099.

12 Hung CS, Dodson KW, Hultgren SJ. A murine model of urinary tract infection. *Nat Protoc* 2009;**4**:1230–43. https://doi.org/10.1038/nprot.2009.116.

13 Price B, Dennison C, Tschesche H, Elliott E. Neutrophil tissue inhibitor of matrix metalloproteinases-1 occurs in novel vesicles that do not fuse with the phagosome. *J Biol Chem* 2000;**275**:28308–15. https://doi.org/10.1074/jbc.M002263200.

14 Schneider CA, Rasband WS, Eliceiri KW. NIH Image to ImageJ: 25 years of image analysis. *Nat Methods* 2012;**9**:671–5. https://doi.org/10.1038/nmeth.2089.

15 Stilley JAW, Birt JA, Nagel SC, Sutovsky M, Sutovsky P, Sharpe-Timms KL. Neutralizing TIMP1 restores fecundity in a rat model of endometriosis and treating control rats with TIMP1 causes anomalies in ovarian function and embryo development. *Biol Reprod* 2010;**83**:185–94. https://doi.org/10.1095/biolreprod.109.083287.

16 Hollmén M, Karaman S, Schwager S, Lisibach A, Christiansen AJ, Maksimow M, *et al.* GCSF regulates macrophage phenotype and associates with poor overall survival in human triple-negative breast cancer. *Oncoimmunology* 2016;**5**:1–17. https://doi.org/10.1080/2162402X.2015.1115177.

17 Zheng GXY, Terry JM, Belgrader P, Ryvkin P, Bent ZW, Wilson R, *et al.* Massively parallel digital transcriptional profiling of single cells. *Nat Commun* 2017;**8**:. https://doi.org/10.1038/ncomms14049.

18 Butler A, Hoffman P, Smibert P, Papalexi E, Satija R. Integrating single-cell transcriptomic data across different conditions, technologies, and species. *Nat Biotechnol* 2018;**36**:411–20. https://doi.org/10.1038/nbt.4096.

19 Phipson B, Sim CB, Porrello ER, Hewitt AW, Powell J, Oshlack A. Propeller: Testing for Differences in Cell Type Proportions in Single Cell Data. *Bioinformatics* 2022;**38**:4720–6. https://doi.org/10.1093/bioinformatics/btac582.

20 Stein T, Morris JS, Davies CR, Weber-Hall SJ, Duffy MA, Heath VJ, *et al.* Involution of the mouse mammary gland is associated with an immune cascade and an acute-phase response, involving LBP, CD14 and STAT3. *Breast Cancer Res* 2004;**6**:75–91. https://doi.org/10.1186/bcr753.

21 Bartoschek M, Oskolkov N, Bocci M, Lövrot J, Larsson C, Sommarin M, *et al.* Spatially and functionally distinct subclasses of breast cancer-associated fibroblasts revealed by single cell RNA sequencing. *Nat Commun* 2018;**9**:. https://doi.org/10.1038/s41467-018-07582-3.

22 Kanaya N, Chang G, Wu X, Saeki K, Bernal L, Shim HJ, *et al.* Single-cell RNA-sequencing analysis of estrogen- and endocrine-disrupting chemical-induced reorganization of mouse mammary gland. *Commun Biol* 2019;**2**:1–15. https://doi.org/10.1038/s42003-019-0618-9.

23 Jin S, Guerrero-Juarez CF, Zhang L, Chang I, Ramos R, Kuan CH, *et al.* Inference and analysis of cell-cell communication using CellChat. *Nat Commun* 2021;**12**:1–20. https://doi.org/10.1038/s41467-021-21246-9.

24 Cordenonsi M, Zanconato F, Azzolin L, Forcato M, Rosato A, Frasson C, *et al.* The hippo transducer TAZ confers cancer stem cell-related traits on breast cancer cells. *Cell* 2011;**147**:759–72. https://doi.org/10.1016/j.cell.2011.09.048.

25 Guo Q, Sun D, Barrett AS, Jindal S, Pennock ND, Conklin MW, *et al.* Mammary collagen is under reproductive control with implications for breast cancer. *Matrix Biol* 2022;**105**:104–26. https://doi.org/10.1016/j.matbio.2021.10.006.

26 Neeli I, Khan SN, Radic M. Histone Deimination As a Response to Inflammatory Stimuli in Neutrophils. *J Immunol* 2008;**4**:.
