# Supplementary material for: Host response during unresolved urinary tract infection alters female mammary tissue homeostasis through collagen deposition and TIMP1
Source: Nat Commun. 2024 Apr 16;15:3282. doi: 10.1038/s41467-024-47462-7 (PMC11021735; doi:10.1038/s41467-024-47462-7)
Supplement: Supplementary file 3 — Description of Additional Supplementary Files [file 41467_2024_47462_MOESM3_ESM.pdf]

Description of Additional Supplementary Files

Supplementary Data 1. Log2FC and p-values for genes included in the YAP signaling signature in NP Epithelial Clusters (Fig. 2C); avg_log2FC: log fold-change of the average expression between two groups, positive values indicate that that feature is highly expressed in defined cluster and condition, negative values indicate that that feature is lowly expressed in defined cluster and condition.

Supplementary Data 2. Log2FC and p-values for genes included in the MEC-ECM Communication Signature in NP Epithelial Clusters (Fig. S3A); avg_log2FC: log fold-change of the average expression between two groups, positive values indicate that that feature is highly expressed in defined cluster and condition, negative values indicate that that feature is lowly expressed in defined cluster and condition.

Supplementary Data 3. Log2FC and p-values for genes included in the mechano-signaling signature in NP Epithelial Clusters (Fig. S3B); avg_log2FC: log fold-change of the average expression between two groups, positive values indicate that that feature is highly expressed in defined cluster and condition, negative values indicate that that feature is lowly expressed in defined cluster and condition.

Supplementary Data 4. Log2FC and p-values for genes included in the YAP signaling signature in PLI Epithelial Clusters (Fig. 3G); avg_log2FC: log fold-change of the average expression between two groups, positive values indicate that that feature is highly expressed in defined cluster and condition, negative values indicate that that feature is lowly expressed in defined cluster and condition.

Supplementary Data 5. Log2FC and p-values for genes included in the Neutrophil Recruitment Signature in NP Epithelial Clusters (Fig. 4A); avg_log2FC: log fold-change of the average expression between two groups, positive values indicate that that feature is highly expressed in

defined cluster and condition, negative values indicate that that feature is lowly expressed in defined cluster and condition.

Supplementary Data 6. Log2FC and p-values for genes included in the Neutrophil Recruitment Signature in PLI Epithelial Clusters (Fig. S6A); avg_log2FC: log fold-change of the average expression between two groups, positive values indicate that that feature is highly expressed in defined cluster and condition, negative values indicate that that feature is lowly expressed in defined cluster and condition.
