# Supplementary material for: Host response during unresolved urinary tract infection alters female mammary tissue homeostasis through collagen deposition and TIMP1
Source: Nat Commun. 2024 Apr 16;15:3282. doi: 10.1038/s41467-024-47462-7 (PMC11021735; doi:10.1038/s41467-024-47462-7)
Supplement: Supplementary file 10 — Reporting Summary [file 41467_2024_47462_MOESM10_ESM.pdf]

nature portfolio

Corresponding author(s): dos Santos, Camila

Last updated by author(s): Mar 14, 2024

Reporting Summary

Nature Portfolio wishes to improve the reproducibility of the work that we publish. This form provides structure for consistency and transparency in reporting. For further information on Nature Portfolio policies, see our Editorial Policies and the Editorial Policy Checklist.

Statistics

For all statistical analyses, confirm that the following items are present in the figure legend, table legend, main text, or Methods section.

n/a	Confirmed	
☐	☒	The exact sample size (n) for each experimental group/condition, given as a discrete number and unit of measurement
☐	☒	A statement on whether measurements were taken from distinct samples or whether the same sample was measured repeatedly
☐	☒	The statistical test(s) used AND whether they are one- or two-sided<br>*Only common tests should be described solely by name; describe more complex techniques in the Methods section.*
☐	☒	A description of all covariates tested
☐	☒	A description of any assumptions or corrections, such as tests of normality and adjustment for multiple comparisons
☐	☒	A full description of the statistical parameters including central tendency (e.g. means) or other basic estimates (e.g. regression coefficient) AND variation (e.g. standard deviation) or associated estimates of uncertainty (e.g. confidence intervals)
☐	☒	For null hypothesis testing, the test statistic (e.g. F, t, r) with confidence intervals, effect sizes, degrees of freedom and P value noted<br>*Give P values as exact values whenever suitable.*
☒	☐	For Bayesian analysis, information on the choice of priors and Markov chain Monte Carlo settings
☐	☒	For hierarchical and complex designs, identification of the appropriate level for tests and full reporting of outcomes
☒	☐	Estimates of effect sizes (e.g. Cohen's d, Pearson's r), indicating how they were calculated

Our web collection on statistics for biologists contains articles on many of the points above.

Software and code

Policy information about availability of computer code

Data collection	Aperio Light Field Slide Scanner (Leica Biosystems), Aperio ImageScope (Leica Biosystems), Zeiss LSM710 confocal microscope with Zen 2012 SP5 software (Zeiss), Zeiss Axio Observer inverted fluorescence microscope using Zen blue 2.0 software (Zeiss), BD LSRFortessa Dual SORP (BD Biosciences), FACSDiva™ v9 software (BD Biosciences), SpectraMax i3x Multi-Mode Microplate Detection Platform (Molecular Devices), 10X Chromium System (10X Genomics), NextSeq 550 High Output Sequencer (Illumina),
Data analysis	FIJI (ImageJ) , CT-FIRE (University of Michigan-Wisconsin), MATLAB (MathWorks), FlowJo™ v10 software (BD Life Sciences), SoftMax Pro software (Molecular Devices), Cell Ranger v.3.1.0 (10X Genomics), Seurat v.4.1.1 , UCell package, Propeller from the Speckle package, Gene Set Enrichment Analysis (GSEA) v4.1, CellChat package, GraphPad Prism 7, gplots (for heatmaps)

For manuscripts utilizing custom algorithms or software that are central to the research but not yet described in published literature, software must be made available to editors and reviewers. We strongly encourage code deposition in a community repository (e.g. GitHub). See the Nature Portfolio guidelines for submitting code & software for further information.

Data

Policy information about availability of data

All manuscripts must include a data availability statement. This statement should provide the following information, where applicable:

- Accession codes, unique identifiers, or web links for publicly available datasets
- A description of any restrictions on data availability
- For clinical datasets or third party data, please ensure that the statement adheres to our policy

scRNA-seq were deposited into BioProject database under number PRJNA855880, and will be publicly available as of the date of publication. Results datasets used on Fig. 2 and Fig. S3 ('no-UTI' -seq) were previously deposited into BioProject database number PRJNA677888. All accession numbers are listed in Supp. Data 11. Source data are provided with this paper. Code and featured feature matrixes are available at https://github.com/dosSantosLabCSHL/scRNA-NP-P-UTI-SC-2022.

Human research participants

Policy information about studies involving human research participants and Sex and Gender in Research.

Reporting on sex and gender	n/a
Population characteristics	n/a
Recruitment	n/a
Ethics oversight	n/a

Note that full information on the approval of the study protocol must also be provided in the manuscript.

Field-specific reporting

Please select the one below that is the best fit for your research. If you are not sure, read the appropriate sections before making your selection.

☒ Life sciences ☐ Behavioural & social sciences ☐ Ecological, evolutionary & environmental sciences

For a reference copy of the document with all sections, see nature.com/documents/nr-reporting-summary-flat.pdf

Life sciences study design

All studies must disclose on these points even when the disclosure is negative.

Sample size

Sample size was determined based on reported size of previous studies that investigated: mammary gland analysis (n=3 or more), and single cell RNAseq analysis (n=2 or more), according to following publications:

dos Santos, C.O., et al., Molecular hierarchy of mammary differentiation yields refined markers of mammary stem cells. Proc Natl Acad Sci U S A, 2013. 110(18): p. 7123-30.

Henry, S., Trousdell, M.C., Cyrill, S.L., Feigman, M.J., Bouhuis, J.M., Aylard D.A., dos Santos C.O. Characterization of gene expression signatures for the identification of cellular heterogeneity in the developing mammary gland. (2021) Journal of Mammary Gland and Neoplasia. PMID: 33988830

Feigman, M., Moss, M., Chen, C., Cyrill,S., Trousdell, M., Frey, W., Yang, S.T., Wilkison, E.J., dos Santos, C.O. Pregnancy reprograms the enhancer landscape of mammary epithelial cells and blocks the development of premalignant lesion. Nature Communications. 11, 2649 (2020). https://doi.org/10.1038/s41467-020-16479-z

Hanasogue Somasundara, A. V., Moss, M.A., Feigman, M.J., Chen, C., Cyrill, S.L., Ciccone, M.F., Trousdell, M.C., Vollbrecht M., S. Li., Kendall J., Beyaz, S., Wilkinson, J.E., dos Santos, C.O. Parity-induced changes to mammary epithelial cells control NKT cell expansion and mammary oncogenesis. (2021). Cell Reports, 37(10):110099. PMID: 34879282

Schedin P, Keely PJ. Mammary gland ECM remodeling, stiffness, and mechanosignaling in normal development and tumor progression. Cold Spring Harb Perspect Biol 2011;3:1–22. https://doi.org/10.1101/cshperspect.a003228.

Wang QA, Scherer PE. Remodeling of Murine Mammary Adipose Tissue during Pregnancy, Lactation, and Involution. J Mammary Gland Biol Neoplasia 2019;24:207–12.https://doi.org/10.1007/s10911-019-09434-2.

Kanaya N, Chang G, Wu X, Saeki K, Bernal L, Shim HJ, et al. Single-cell RNA-sequencing analysis of estrogen- and endocrine-disrupting chemical-induced reorganization of mouse mammary gland. Commun Biol 2019;2:1–15. https://doi.org/10.1038/s42003-019-0618-9.

Data exclusions	no data was excluded
Replication	at least 3 replicates per question - all attempts to repeat the experiment and analysis were successful
Randomization	The experimental analysis described on this manuscript were preformed in healthy or UTI-bearing mice, thus no randomization was performed for these analysis. UTI-bearing animals were randomized prior their treatment with anti-CSF-3 or anti-TIMP1 neutralizing antibodies. Covariates such as mouse age, and UTI infection were controlled to remove effects of variables across the experimental approaches.
Blinding	The studies presented in this manuscript were not blinded.

Behavioural & social sciences study design

All studies must disclose on these points even when the disclosure is negative.

Study description	n/a
Research sample	n/a
Sampling strategy	n/a
Data collection	n/a
Timing	n/a
Data exclusions	n/a
Non-participation	n/a
Randomization	n/a

Ecological, evolutionary & environmental sciences study design

All studies must disclose on these points even when the disclosure is negative.

Study description	n/a
Research sample	n/a
Sampling strategy	n/a
Data collection	n/a
Timing and spatial scale	n/a
Data exclusions	n/a
Reproducibility	n/a
Randomization	n/a
Blinding	n/a

Did the study involve field work? ☐ Yes ☒ No

Reporting for specific materials, systems and methods

We require information from authors about some types of materials, experimental systems and methods used in many studies. Here, indicate whether each material, system or method listed is relevant to your study. If you are not sure if a list item applies to your research, read the appropriate section before selecting a response.

Materials & experimental systems

n/a	Involved in the study
	☒ Antibodies
☒	Eukaryotic cell lines
☒	Palaeontology and archaeology
	☒ Animals and other organisms
☒	Clinical data
☒	Dual use research of concern

Methods

n/a	Involved in the study
☒	ChIP-seq
	☒ Flow cytometry
☒	MRI-based neuroimaging

Antibodies

Antibodies used

All antibodies were obtained from commercial vendors and used without further purification. For immunofluorescence staining: Alexa Fluor 647-conjugated anti-Cytokeratin 5 (EP1601Y, Abcam, cat# ab193895, 0.5 mg/ml, 1:200), Alexa Fluor 488-conjugated anti-β-casein (H-4, SCBT, cat# sc-166530, 0.2 mg/ml, 1:200), FITC-conjugated anti-E. coli antibody (Abcam, cat# ab30522, 5mg/ml, 1:100), anti-human/mouse myeloperoxidase antibody (R&D Systems, cat# AF3667, 0.2 mg/ml, 1:100), Anti-Histone H3 (citrulline R2 + R8 + R17) antibody (Abcam, cat# ab5103, 1 mg/ml, 1:250), Alexa Fluor 568-conjugated anti-goat secondary antibody (Invitrogen, cat# A10037, 2 mg/ml, 1:150) and Alexa Fluor 488-conjugated anti-rabbit secondary antibody (Invitrogen, cat# A21206, 2 mg/ml, 1:150, Picrosirius Red staining kit (Abcam, cat# ab150681), anti-Perilipin 1 polyclonal antibody (Thermo Fisher, cat# PA5-55046, 1:200), Alexa Fluor 488-conjugated anti-Fibronectin (Abcam cat# ab237286, 1:200). For flow cytometry: Brilliant Violet 605-conjugated anti-CD45 antibody (30-F11, BioLegend, cat# 103140, 0.2 mg/ml, 3:200), PE/Cy7-conjugated anti-CD11b antibody (M1/70, BioLegend, cat# 101216, 0.2 mg/ml, 1:100) and, Alexa Flour 700-conjugated anti-Ly6G antibody (1A8, BioLegend, cat# 127622, 0.5 mg/ml, 3:200).

Validation

Describe the validation of each primary antibody for the species and application, noting any validation statements on the manufacturer's website, relevant citations, antibody profiles in online databases, or data provided in the manuscript.

Animals and other research organisms

Policy information about studies involving animals; ARRIVE guidelines recommended for reporting animal research, and Sex and Gender in Research

Laboratory animals

Nulliparous and timed-pregnant (gestation day E11-E15), female, C57BL/6J mice were purchased from The Jackson Laboratory. All animals were housed at the CSHL shared Laboratory Animal Resource under a 12 hr. light/dark cycle, with controlled temperature and humidity at 72oF and 40-60%, respectively, and with access to dry food and water ad libitum, unless otherwise specified.

Wild animals

This study did not involve wild animals

Reporting on sex

The experiments were performed in female mice only, given the developed state of mammary tissue

Field-collected samples

This study did not involve field-collected samples

Ethics oversight

All animal experiments were performed in accordance with CSHL Institutional Animal Care and Use Committee guidelines.

Note that full information on the approval of the study protocol must also be provided in the manuscript.

Flow Cytometry

Plots

Confirm that:

☒ The axis labels state the marker and fluorochrome used (e.g. CD4-FITC).

☒ The axis scales are clearly visible. Include numbers along axes only for bottom left plot of group (a 'group' is an analysis of identical markers).

☒ All plots are contour plots with outliers or pseudocolor plots.

☒ A numerical value for number of cells or percentage (with statistics) is provided.

Methodology

Sample preparation

Mammary glands were digested into single cell suspensions using previously published protocols9–11. Briefly, inguinal and thoracic mammary glands were harvested, minced and digested for ~90 min at 37oC in RPMI 1640 GlutaMAX (Gibco, cat# 61870127) containing 5% FBS (Corning, cat# 35-010-CV) and 1X Collagenase-Hyaluronidase (Stem Cell Technology, cat# 07912). Digested mammary glands were centrifuged at 2000 rpm for 5 mins and the pellet was cryopreserved in 1 ml of Synth-a-Freeze™ Cryopreservation Medium (Gibco, cat# A1254201), distributed over two cryovials (Corning, cat# 430487), and stored at -80oC for short-term storage and in liquid nitrogen vapor for long-term storage. Viably frozen, digested mammary fragments were thawed in a 37oC water bath, and washed with chilled HBSS (Gibco, cat# 14175103) containing 5%

FBS. Single cell suspensions were obtained by incubating the cell pellet with 3 ml of TrypLE Express (Gibco, cat# 12604013) for 3 min followed by an HBSS wash, and subsequent incubation with 1 ml of Dispase (Stem Cell Technology, cat# 07913) containing 40µl DNase I (Sigma, cat# D4263) for 2 minutes. The cell suspension was washed again in HBSS and filtered through a 100 µm cell strainer (BD Falcon, cat# c352360). Centrifugation at 2000 rpm for 5 min at room temperature was used to collect cells after each wash. The cells were resuspended in 1X MACS buffer (1X PBS with 0.5% FBS) and kept on ice prior to staining for flow cytometry. Single cells suspended in 1X MACS buffer were stained with appropriate fluorophore-conjugated primary antibodies. Staining was carried out in a 5 ml polystyrene round-bottom tube (Corning, cat# 352054) for 40 min at 4oC in the dark after which the samples were washed in 1X MACS buffer and transferred to a polystyrene round bottom tube fitted with a 35 µm cell strainer cap (Corning, cat# 352235) prior to acquisition. zx

Instrument

BD LSRFortessa Dual SORP (BD Biosciences)

Software

BD FACSDiva™ v9 software (BD Biosciences), FlowJo™ v10 software (BD Life Sciences)

Cell population abundance

Mammary resident/infiltrating neutrophils accounted for ~10% of singlet population defined from SSC_H x SSC_W and FSC_H x FSC_W gates. Circulating immune cells (blood samples) account for ~20-30% of singlet populations defined from SC_H x SSC_W and FSC_H x FSC_W gates. Bone marrow derived granulocytes and monocytes (in vitro analysis) account for 4%-30% of singlets define from SC_H x SSC_W and FSC_H x FSC_W gates.

Gating strategy

For datasets that utilized FACS-sorted MECs, gating strategy was utilized as previously published:

Hanasogue Somasundara, A. V., Moss, M.A., Feigman, M.J., Chen, C., Cyrill, S.L., Ciccone, M.F., Trousdell, M.C., Vollbrecht M., S. Li., Kendall J., Beyaz, S., Wilkinson, J.E., dos Santos, C.O. Parity-induced changes to mammary epithelial cells control NKT cell expansion and mammary oncogenesis. (2021). Cell Reports, 37(10):110099. PMID: 34879282

Nowroozilarki N, Öz HH, Schroth C, Hector A, Nürnberg B, Hartl D, et al. Anti-inflammatory role of CD11b+Ly6G+ neutrophilic cells in allergic airway inflammation in mice. Immunol Lett 2018;204:67–74. https://doi.org/10.1016/j.imlet.2018.10.007.

Tam JW, Kullas AL, Mena P, Bliska JB, Van der Velden AWM. CD11b+ Ly6Chi Ly6Gimmatur myeloid cells recruited in response to Salmonella enterica serovar typhimurium infection exhibit protective and immunosuppressive properties. Infect Immun 2014;82:2606–14. https://doi.org/10.1128/IAI.01590-13.

☒ Tick this box to confirm that a figure exemplifying the gating strategy is provided in the Supplementary Information.
